# Evolutionary pathways to convergence in plumage patterns

**DOI:** 10.1186/s12862-016-0741-x

**Published:** 2016-08-31

**Authors:** Thanh-Lan Gluckman, Nicholas I. Mundy

**Affiliations:** 1 Department of Zoology, University of Cambridge, Downing Street, Cambridge, CB2 3EJ UK; 2 Department of Animal and Plant Sciences, University of Sheffield, Alfred Denny Building, Western Bank, Sheffield, S10 2TN UK; 3 Center for Interdisciplinary Research in Biology, College de France, Paris, 75005 France

**Keywords:** Within-feather patterning, Evo-devo, Modularity, Comparative modelling

## Abstract

**Background:**

Avian plumage is ideal for investigating phenotypic convergence because of repeated evolution of the same within-feather patterns. In birds, there are three major types of regular patterns within feathers: scales, bars and spots. Existing models of within-feather pattern development suggest that scales have the simplest developmental mechanism, bars require more stringent regulation than scales, and spots have the strictest developmental parameters. We hypothesized that increasing stringency in the mechanism of pattern formation predicts the evolutionary trajectory of patterns, and hence scales should evolve first, followed by bars and finally spots. Here, using Bayesian phylogenetic modeling we reconstructed pattern evolution in the most spectacularly patterned avian clades – aquatic waterfowl (Anseriformes) and terrestrial gamebirds (Galliformes).

**Results:**

Our analyses suggest that the ancestral state of plumage is an absence of patterns, but with some variability. Independent analyses of seven feather patches reveal that spots evolve after bars and scales. However, both scales and bars evolve frequently from an absence of patterns, contradicting our predictions. Over the whole body, many constraints are conserved from the level of patches, for example the largest number of steps from the ancestral state was required for spots to evolve.

**Conclusions:**

Overall there was remarkable similarity in the inferred evolutionary trajectories of plumage pattern evolution in Galliformes and Anseriformes, suggesting that developmental constraint is similar in these two orders, despite large ecological differences. These evolutionary transitions are largely congruent with a reaction–diffusion based model of pattern formation, but the evolution of bars from an unpatterned ancestor is more common than expected. Our study highlights the promise of testing models of development using comparative methods.

**Electronic supplementary material:**

The online version of this article (doi:10.1186/s12862-016-0741-x) contains supplementary material, which is available to authorized users.

## Background

Comparative studies are a powerful tool for understanding the underlying processes behind similarity in animal forms and have revealed that the mechanisms underlying convergent evolution are diverse as well as surprising [1, 2]. Phenotypic convergence may arise from similar selective regimes, but may also be at least partly explained by developmental constraint [3–5]. Under developmental constraint, some phenotypes are developmentally more readily accessible than others, thereby biasing evolution to follow particular pathways.

**Fig. 1:**
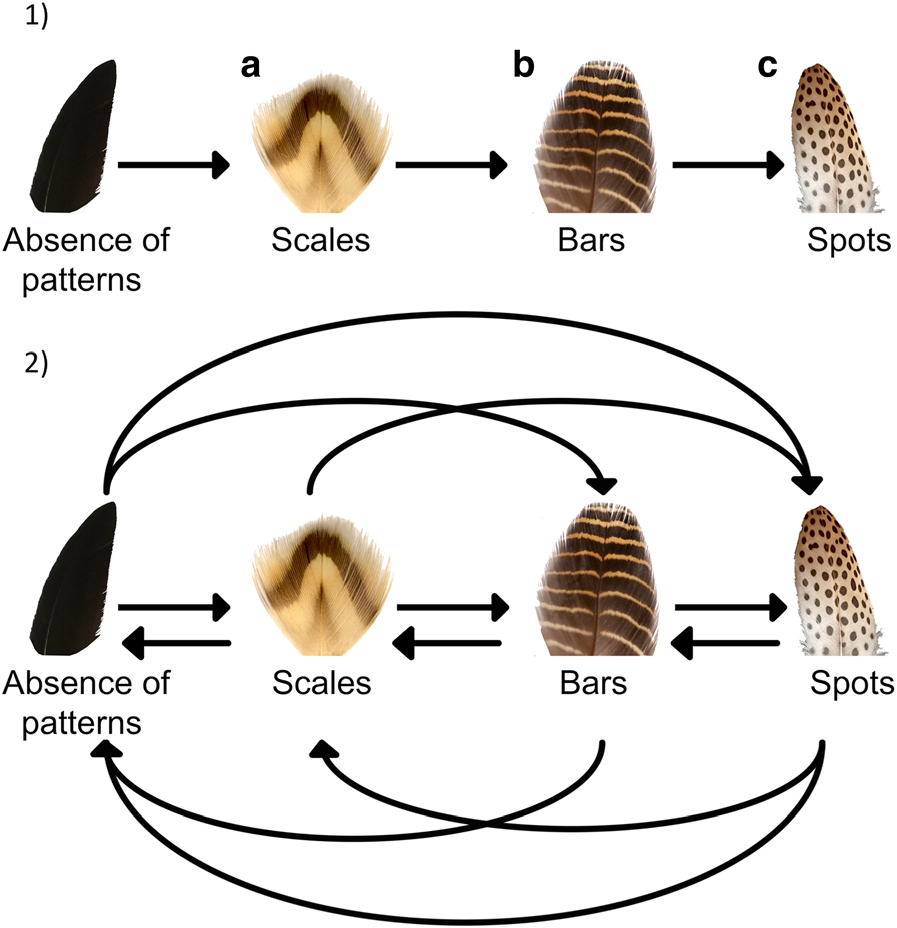
Hypothesis of developmental constraint in plumage pattern evolution on the basis of increasing complexity and the null (full) model of pattern evolution. 1) Model of developmental constraint: 1.1a) Scales - king eider (*Somateria spectabilis*), 1.1b) Bars - snow partridge (*Lerwa lerwa*), 1.1c) Spots - great argus (*Argusianus argus*). If there is developmental constraint in plumage pattern evolution on the basis of increasing stringency, then perhaps scales evolve from the ancestral state of uniform coloration, followed by bars, and finally spots. 2) The null model of plumage pattern evolution, also known as the full model, where there is no directionality and all evolutionary transitions occur. Images were taken at the University Museum of Zoology, the University of Cambridge, by T-L. Gluckman and are copyright of the University Museum of Zoology

**Fig. 2:**
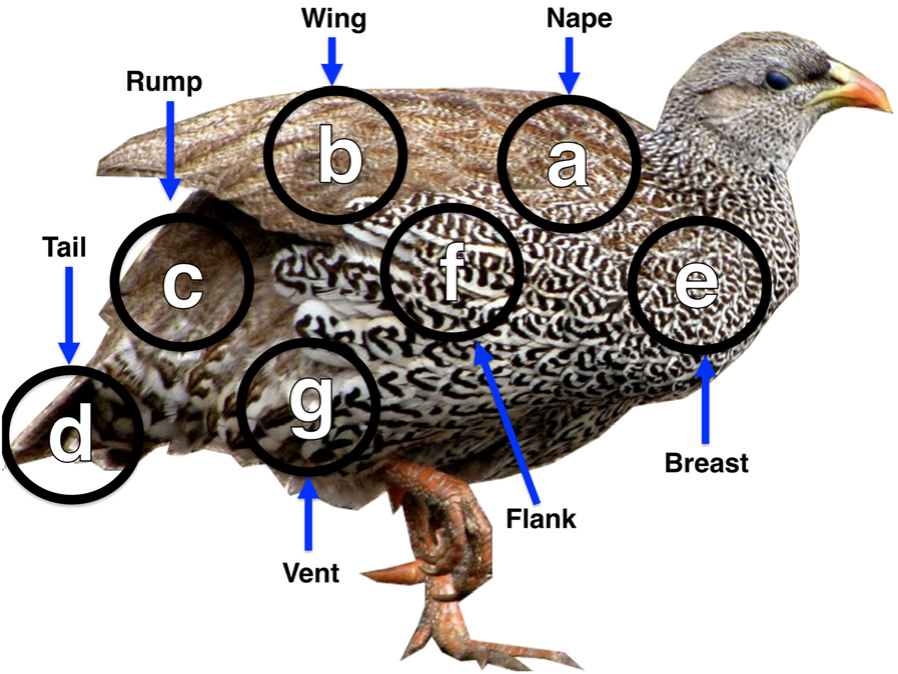
The seven individual plumage patches sampled in this study. 1) Field guide terminology, and the corresponding feather tracts (pterylae; [18]) are as follows: a) Nape: interscapular tract, b) Wing (scapular, wing coverts, tertials, primaries and secondaries): humeral tract, upper marginal coverts of prepatagium and upper wing covert tract, c) Rump and uppertail coverts: dorsopelvic tract and dorsal caudal tract, d) Tail: upper major tail covert, upper median tail covert and rectrices tract, e) Breast: ventral cervical tract, f) Flanks or side: pecterosternal tract in Anseriformes or pectoral tract in Galliformes, g) Vent and undertail coverts: abdominal tract in Anseriformes or lateral and medial abdominal tracts in Galliformes. 2) In the whole body analysis, all patches are analysed together. The species illustrated is the Natal francolin (*Pternistes francolinus*)

The spectacular plumages of birds have been subject to considerable attention due to their diversity, functional significance and ease of study e.g. [6–10]. Plumage patches may be comprised of uniformly coloured or patterned feathers (Fig. 1). While the evolution of plumage coloration has been extensively studied, the evolution of within-feather patterning has received less attention despite abundant interspecific variation [9, 11]. Plumage variation over the body can occur via variation in feather patterning among patches, which generally correspond to the sub-compartments of the major feather tracts (pterylae) (Fig. 2) [12]. For example, the tail of the peacock (*Pavo cristatus*) has coloured spots whereas the wings have bars.

Within-feather patterning can be split into two types based on the distribution of pigmentation: irregular pigmentation (mottled plumage), where the vane is heterogeneously pigmented, and regular patterns, which are comprised of the same recurring motif [13, 14]. Regular within-feather patterns have largely converged on three simple repeating geometric patterns: scales - where the feather border is coloured differently (Fig. 1a); bars - alternating bars of lighter and darker coloration perpendicular to the feather’s axis (Fig. 1b); and spots - one or more spots within feathers (Fig. 1c) (Fig. 1*in* [13]). A few other regular feather patterns occur rarely, including chevrons, longitudinal stripes, and checkered patterning, such as in the common loon (*Gavia immer*).

Melanins are of key importance in within-feather patterns since they are the only pigments that can be differentially deposited in a precise spatio-temporal sequence during feather growth [13, 14]. In contrast, carotenoid-based coloration is typically confined to feather tips and so only contributes to uniform patches of coloration over the body. In a comparative survey encompassing 90 % of avian species there were no observed cases of plumage patterns where melanin appeared to be absent, and additional types of coloration in patterning (e.g. psittacofulvins and carotenoids) were rare (T-L. Gluckman unpublished data).

Development of feather patterns has been modelled using Turing reaction–diffusion models. Turing systems form morphological patterns from the combined action of two molecules (morphogens), an activator and an inhibitor. Chemical gradients of the morphogens induce spatially explicit patterns that are controlled by four key parameters per morphogen: the rates of production, decay and diffusion, as well as strength of their interaction [15].

A few studies have investigated patterning among different feathers or hairs using reaction–diffusion models. Price and Pavelka [16] studied pattern evolution at the level of different patches, and showed that the evolution of patches of white plumage (unmelanized feathers) in leaf warblers (*Phylloscopus*) could be attributed to increasing and decreasing rates of morphogen production. For mammalian coloration, the application of Turing models could explain why mammals that have a spotted tail with a striped body are not found [17], thus implying a form of developmental constraint, best considered as a relative constraint [16].

In a landmark study, all regular within-feather patterns were successfully simulated with a reaction–diffusion based model [13]. By modeling differential pigment uptake by keratinocytes during feather development, Prum and Williamson varied the spatial and temporal periodicity of pigmentation dynamics to produce the most commonly found regular plumage patterns. According to this model, the production of scales has a low rate of morphogen decay and is governed by spatial periodicity of melanin uptake. The production of bars requires a higher rate of morphogen decay resulting in temporal periodicity of melanin uptake. Notably, the formation of spots is distinct as it is comprised of simultaneous spatial and temporal differentiation and has the narrowest range of parameters (Figure 6 in Prum and Williamson *in* [13]).

From Prum and Williamson’s model [13], we hypothesised that the mechanism of within-feather pattern formation may bias the production of pattern variation during evolution in a stepwise order from decreasing to increasing stringency, thereby acting as a constraint. Our interpretation of this model of within-feather pattern formation is that scales have the least stringent conditions, bars have more stringent parameters than scales, and spots have the narrowest range of parameters. Therefore, our hypothesis of within-feather pattern evolution is that scales evolved first, followed by bars and finally spots (Fig. 1.1). Our approach here is to use comparative Bayesian methods to infer the evolutionary pathways of pattern evolution. We can then observe whether the inferred pathways are consistent with our hypothesis of constrained evolution inspired by Prum and Williamson’s reaction–diffusion model [13], providing insights into whether the model is realistic.

From what is currently understood of plumage development, covariation of feather pigmentation within patches is indicative of local shared developmental mechanisms and hence patches are a logical focus to study plumage pattern evolution (Fig. 2) [18–20]. At the level of the whole body, evolution of a novel plumage pattern may occur within the same patch or involve recruitment from other patches, or other modules, and might therefore appear relatively unconstrained. Analysis of the whole body is complicated by the co-occurrence of multiple different pattern types within a species, e.g. species in both Anseriformes (e.g. Hottentot teal, *Anas hottentoti*) and Galliformes (e.g. Elliot’s pheasant, *Syrmaticus ellioti*) have separate patches with all four pattern phenotypes considered here: absence, scales, bars and spots. Scoring of patterns over the whole body thus necessitates prioritizing particular patterns over others.

These considerations lead to the development of a hierarchical approach in which we first consider evolution within patches, then evolution over the whole body. Sequential steps in evolution would demonstrate that some transitions are preferred over others and allow a direct test of our hypotheses. Similarity at the level of the whole body would suggest an absence of other mechanisms at this level.

The avian orders with the most spectacular plumage patterns are the waterfowl (Anseriformes) and gamebirds (Galliformes), which together form a monophyletic group (Galloanserae; [21–24]). Each order includes iconic examples of patterns such as the spotted plumage of the great argus (*Argusianus argus;* Galliformes*,* Fig. 1c). Anseriformes and Galliformes have dramatically different lifestyles, comprising waterbirds and landbirds, respectively [25, 26], and are thus likely to be subject to a host of different selection pressures. Variation in selection pressure is ideal for testing our hypothesis of developmental constraint in these two orders because developmental constraint should lead to similar evolutionary pathways.

Here, we examine whether within-feather pattern evolution follows predictable sequential steps, using Bayesian phylogenetic modeling in Anseriformes and Galliformes separately, with patterning identified from museum skins. We traced pattern evolution in a hierarchical order to assess whether there may be generalities in these ecologically diverse groups of birds and examine whether a) pattern evolution is sequential, b) whether the direction of evolution provides support for increasing stringency in within-feather patterning developmental mechanisms, c) whether the direction is consistent with our interpretation of Prum and Williamson’s reaction–diffusion model, d) whether convergence follows similar pathways in both orders, and e) whether global models of plumage pattern evolution differ from the developmental models of within patches.

## Results

### Taxonomic distribution of patterns

All of the different types of regular plumage patterns were represented in the seven plumage patches, with the exception of spots on the rump and tail, as well as bars on the tail in Anseriformes, and scales on the tail in Galliformes (Tables 1 and 2; *see* Additional file 1: Figure S1 & S2 for a taxonomic distribution of plumage patterns). We first present analyses of plumage pattern evolution within individual patches followed by the whole body. All models are presented in two ways to account for phylogenetic uncertainty: models obtained using branch length information as supplied (*herein* all species), and a more robust analysis using only branches with high probability (*herein* robust) (*see* Methods).

**Table 1:** The frequency of the different types of patterns in the seven plumage patches over the body, the number of unique models in the entire posterior sample distribution as well as the top model set, and the average probability and marginal probability of the ancestral state of patterns, in Anseriformes and Galliformes using the full phylogeny. The tail of Anseriformes only has seven species with scaled patterns and was removed from the analyses

	Plumage pattern frequency					Unique models			Average probability				Marginal probability			
	Absence of patterns	Scales	Bars	Spots	Mottled	Posterior sample	Top model set (BF > =2)	Full model	Absence of patterns	Scales	Bars	Spots	Absence of patterns	Scales	Bars	Spots
Anseriformes																
Nape	78	16	19	3	2	464	257	118: BF = 0.08	0.31	0.25	0.20	0.24	0.02; 0.78	0.80; 0.01	0.79; 0.01	0.80; 0.00
Wing	105	5	5	1	2	698	454	57: BF = 0.04	0.27	0.26	0.23	0.23	0.11; 0.67	0.75; 0.03	0.70; 0.08	0.77; 0.01
Rump	100	12	4	-	2	15	4	1435: BF = 0.72	0.33	0.33	0.33	N/A	0.30; 0.38	0.38; 0.29	0.68; 0.00	N/A
Tail	109	7	-	-	2	1	1	All	0.5	0.5	N/A	N/A	0.50; 0.50	0.50; 0.50	N/A	N/A
Breast	74	14	12	13	5	379	218	207: BF = 0.14	0.25	0.27	0.22	0.26	0.44; 0.36	0.61; 0.19	0.77; 0.03	0.65; 0.15
Flanks	65	11	34	5	3	339	164	68: BF = 0.05	0.26	0.25	0.24	0.25	0.12; 0.76	0.88; 0.00	0.77; 0.11	0.88; 0.00
Vent	86	9	14	4	5	379	229	106: BF = 0.07	0.2	0.25	0.29	0.26	0.45; 0.34	0.72; 0.07	0.50; 0.29	0.70; 0.09
Whole body	51	24	26	15	2	142	88	98; BF = 0.34	0.3	0.24	0.27	0.2	0.11; 0.75	0.84; 0.02	0.79; 0.08	0.85; 0.01
Galliformes																
Nape	75	18	29	10	38	229	121	11: BF = 0.01	0.72	0.13	0.11	0.05	0.01; 0.93	0.94; 0.00	0.94; 0.00	0.94; 0.00
Wing	81	14	13	9	53	475	292	40: BF = 0.03	0.66	0.14	0.13	0.07	0.00; 0.87	0.87; 0.00	0.87; 0.00	0.87; 0.00
Rump	83	11	25	8	43	305	178	12: BF = 0.01	0.8	0.09	0.08	0.03	0.00; 0.93	0.93; 0.00	0.93; 0.00	0.93; 0.00
Tail	93	-	24	8	45	36	7	454: BF = 0.20	0.78	N/A	0.09	0.13	0.08; 0.63	N/A	0.71; 0.00	0.63; 0.08
Breast	105	16	17	13	19	265	134	34: BF = 0.02	0.48	0.19	0.2	0.13	0.00; 0.89	0.89; 0.00	0.89; 0.00	0.89; 0.00
Flanks	79	21	29	14	27	399	235	101: BF = 0.07	0.69	0.16	0.09	0.06	0.05; 0.76	0.78; 0.03	0.80; 0.01	0.81; 0.00
Vent	139	6	11	4	10	312	191	10: BF = 0.01	0.48	0.2	0.23	0.09	0.00; 0.95	0.95; 0.00	0.95; 0.00	0.95; 0.00
Whold body (scales)	58	34	38	14	26	114	76	7: BF = 0.23	0.88	0.05	0.05	0.02	0.00; 0.92	0.92; 0.00	0.92; 0.00	0.92; 0.00
Whole body (spots)	54	23	36	26	26	95	63	6: BF = 0.02	0.84	0.09	0.06	0.01	0.00; 0.92	0.92; 0.00	0.92; 0.00	0.92; 0.00

**Table 2:** The frequency of the different types of patterns in the seven plumage patches over the body, the number of unique models in the entire posterior sample distribution as well as the top model set, and the average probability and marginal probability of the ancestral state of patterns, in Anseriformes and Galliformes using branches that have BI = > 0.95. The tail only has seven species with scaled patterns and was removed from the analyses

									Ancestral state							
	Plumage pattern frequency					Unique models			Average probability				Marginal probability			
	Absence of patterns	Scales	Bars	Spots	Mottled	Posterior sample	Top model set (BF > =2)	Full model	Absence of patterns	Scales	Bars	Spots	Absence of patterns	Scales	Bars	Spots
Anseriformes																
Nape	58	12	15	2	1	943	480	662: BF = 0.06	0.30	0.25	0.21	0.24	0.08; 0.68	0.74; 0.02	0.70; 0.06	0.76; 0.00
Wing	77	5	5	1	1	693	446	56: BF = 0.03	0.27	0.26	0.23	0.23	0.09; 0.71	0.76; 0.04	0.74; 0.06	0.79; 0.01
Rump	75	8	3	0	2	20	5	1821: BF = 0.70	0.33	0.34	0.34	N/A	0.65; 0.00	0.62; 0.02	0.45; 0.20	N/A
Breast	54	13	8	9	3	453	161	38: BF = 0.00	0.03	0.33	0.14	0.51	0.92; 0.04	0.92; 0.04	0.91; 0.05	0.13; 0.83
Flanks	45	11	28	3	0	219	46	12: BF = 0.00	0.23	0.28	0.21	0.28	0.15; 0.83	0.93; 0.06	0.98; 0.00	0.89; 0.09
Vent	64	9	10	2	2	336	84	7: BF = 0.00	0.01	0.28	0.35	0.37	0.98; 0.00	0.98; 0.00	0.22; 0.76	0.76; 0.22
Whole body	35	18	23	10	0	250	96	6: BF = 0.00	0.37	0.21	0.25	0.16	0.04; 0.95	0.97; 0.02	0.99; 0.00	0.97; 0.02
Galliformes																
Nape	51	10	11	4	21	584	265	5: BF = 0.00	0.69	0.11	0.13	0.07	0.14; 0.83	0.92; 0.05	0.89; 0.08	0.96; 0.01
Wing	53	6	3	6	29	974	540	61: BF = 0.01	0.85	0.05	0.06	0.04	0.00; 0.89	0.89; 0.00	0.89; 0.00	0.89; 0.00
Rump	58	6	6	6	20	1195	610	28: BF = 0.00	0.89	0.04	0.04	0.02	0.01; 0.90	0.91; 0.00	0.91; 0.00	0.91; 0.00
Tail	57	0	10	8	24	42	11	1399: BF = 0.07	0.41	N/A	0.26	0.33	0.70; 0.14	N/A	0.42; 0.42	0.54; 0.30
Breast	67	8	6	8	8	678	222	35: BF = 0.00	0.56	0.16	0.13	0.14	0.01; 0.94	0.95; 0.00	0.94; 0.01	0.94; 0.01
Flanks	58	11	9	7	12	761	417	28: BF = 0.00	0.46	0.28	0.19	0.06	0.47; 0.46	0.66; 0.27	0.75; 0.18	0.92; 0.01
Vent	85	4	5	3	3	1197	613	67: BF = 0.01	0.48	0.20	0.19	0.12	0.32; 0.56	0.71; 0.17	0.76; 0.12	0.84; 0.04
Whold body (scales)	44	18	14	8	14	352	136	6: BF = 0.00	0.87	0.05	0.06	0.02	0.01; 0.97	0.98; 0.00	0.97; 0.01	0.98; 0.00
Whole body (spots)	41	13	14	15	14	199	496	58: BF = 0.01	0.83	0.09	0.07	0.02	0.00; 0.94	0.94; 0.00	0.94; 0.00	0.94; 0.00

### Evolution within patches of plumage

For individual patches of plumage that have four pattern states, there was variation in the number of unique models supported in the top model set (Tables 1 and 2). Plumage patches with only three pattern states (Anseriformes: rump; Galliformes: tail) had less variation in the number of unique models in the top model set than plumage patches with four pattern states. Across all analyses there was negligible support for the full model of pattern evolution (Fig. 1.2; Tables 1 and 2).

Comparing the models derived from phylogenies with all species and models derived from phylogenies with only robust branches, the occurrence of transitions rarely changed (Additional file 1: Figure S3). For example, in Anseriformes there was no difference in the models of the belly and the breast, and in Galliformes there was no difference in the models of the nape, wing and rump. In the transitions of some models, using only high probability branches improved the probability of transition, e.g. a transition from scales to spots in the breast of Galliformes changed from 0.03; 0.86 to 0.03; 0.92, and the ancestral state of the rump of Galliformes changed from 0.80 to 0.90 (Table 2).

In the analysis using all species, the ancestral plumage was an absence of patterns in five out of six patches of plumage in Anseriformes (robust Anseriformes: three out of six), and all seven patches in Galliformes (robust Galliformes: six out of seven) (Tables 1 and 2). Three patches in Anseriformes have equivocal support in the all species analysis - rump, breast and vent (robust Anseriformes – rump and flanks). However, the marginal probability (MP), which integrates model support, unlike the average probability, indicated that pattern absence is the most probable ancestral plumage in the rump, breast and vent. In the robust Anseriformes analysis, where an absence of patterns was *not* the ancestral state, the MP did not support an alternative pattern being the ancestral state (Tables 1 and 2).

**Fig. 3:**
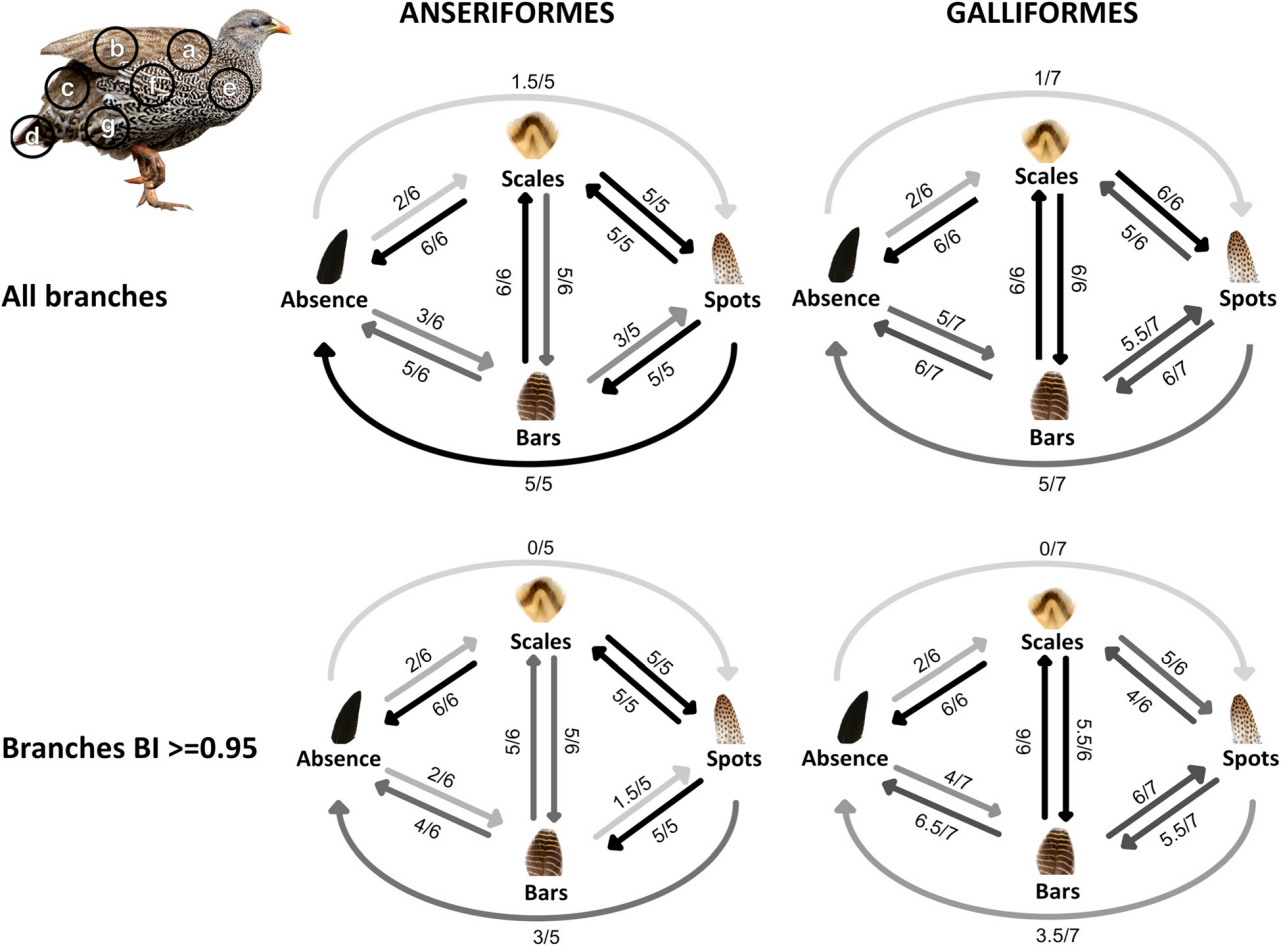
The summary model of local evolution within-patches across all seven plumage patches in Anseriformes and Galliformes. The first panel depicts models derived from unmodified phylogenies and the second panel depicts models derived from phylogenies with only branches with a Bayesian posterior probability = > 0.95 (*see* Methods). Next to each transition is the number of plumage patches in which the transition occurs out of the total number of plumage patches. The total number of plumage patches can vary from the maximum (seven) because in some patches particular patterns do not occur e.g. in Anseriformes no species have evolved spots on the rump, and the tail patch was excluded from the analyses, so the total number of plumage patches in which spots can evolve is five. Where transition lines have an intermediate value, e.g. 1.5/5 for a transition from absence to spots in Anseriformes, this indicates that the transition was equivocal in one of the models of pattern evolution within plumage patches. The weight of each transition probably occurring is represented on a scale of pale grey (occurs rarely) to black (occurs in every plumage patch possible)

In all models of plumage patch evolution, there was evidence for a bias in the direction of evolution as some transitions probably occurred and others did not (Fig. 3, Additional file 1: S2 & S3). Examining the order of pattern evolution within-patches, bars mostly evolve more frequently from an absence of patterns (Anseriformes: 4/6; Galliformes: 5/7) than scales (Anseriformes and Galliformes: 2/6). The exception being Anseriformes robust, where scales or bars evolve from an absence of patterns (Fig. 3). In the majority of models, the average transition rate from an absence of patterns to scales is low (Additional file 1: Figure S2 & S3). In both orders there are strong bidirectional transitions between scales and bars, and spots predominantly evolved from scales (all species - Anseriformes: 5/5, Galliformes: 6/6; robust – Anseriformes: 5/5, Galliformes 5/6) rather than bars (all species - Anseriformes: 3/5; Galliformes: 5.5/6; robust – Anseriformes: 1.5/5, Galliformes: 6/7). Finally, transitions from an absence of patterning to spots were rare and had the lowest rate of transition where they occur (all species - Anseriformes: 1.5/5, Galliformes: 1/7; robust – Anseriformes and Galliformes: 0/5 and 0/7, respectively) (Additional file 1: Figure S2). Therefore, within-patches the main order of plumage pattern evolution is bars first, followed by scales and finally spots.

#### Global model of plumage pattern evolution

**Fig. 4:**
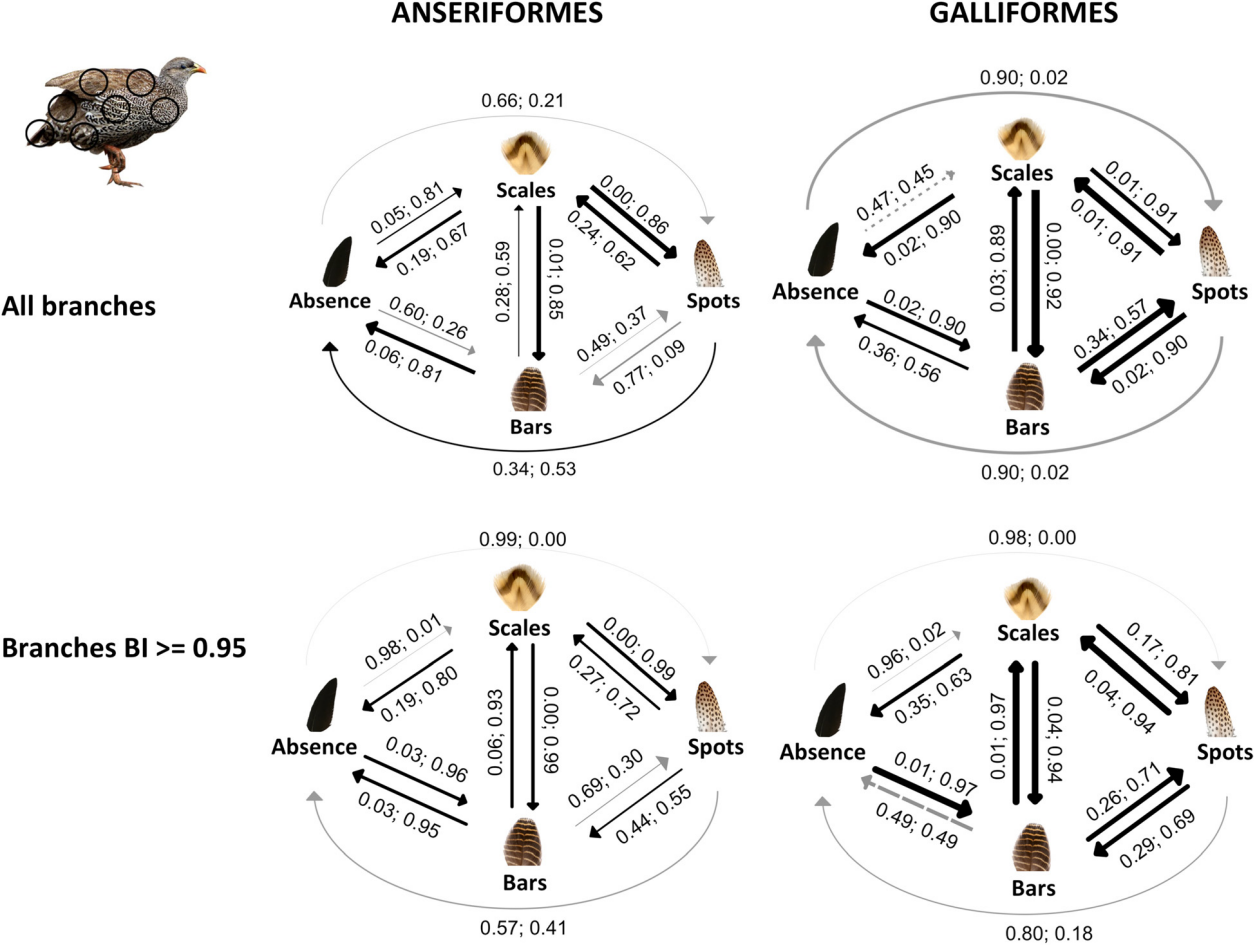
The most probable evolutionary transitions between plumage patterns over the whole body in Anseriformes and Galliformes. Depicted are models where scales evolved first in the robust analysis of Anseriformes, and scales are derived in Galliformes. Next to each transition is the marginal probability of a transition not occurring, followed by the marginal probability of it occurring. The width of the transition line is proportional to the rate of transition. A grey transition line indicates transitions that probably do not occur and black transition lines indicate transitions that probably occur. The marginal probability of occurring and not occurring does not equal due to variation in the top model set

Assigning scales or bars as the first pattern to evolve in Anseriformes, and scales or spots evolving from an existing pattern in Galliformes, resulted in no qualitative differences within models derived from each phylogeny. Furthermore, there was only modest variation in the major transition rates between the global models derived from the all species and robust phylogenies (Additional file 1: Figure S2b & S3b). Therefore we present scales evolving first for the robust analysis of Anseriformes and spots being derived in Galliformes as these are most similar to the summary model of within patches (Fig. 4).

The global models of plumage pattern evolution showed some important similarities with within-patch models. First, in both orders, a direct transition from an absence of patterns to spots probably does not occur. Second, there are strong bidirectional transitions between bars and scales in both orders (Fig. 4, Additional file 1: S3). Third, in Galliformes bars evolve first. However, there were some differences between the global model and the summary model of evolution within-patches: in the Anseriformes global model over all species, scales evolve first and the model lacks bidirectional transitions between bars and spots, but taking into account phylogenetic uncertainty bars appear to have evolved first, there is a transition from spots to bars and there are no bidirectional transitions between an absence of patterns and spots. Similarly, in Galliformes bars evolved first, there are bidirectional transitions between bars and spots, and there are no bidirectional transitions between an absence of patterns and spots (Figs. 3 and 4).

## Discussion

Studies of phenotypic convergence in bird plumage have mostly focused on coloration, although regular patterns within feathers are widespread across the class Aves. From our analyses we suggest that the ancestral state of plumage is an absence of patterns, a consistent finding in Galliformes, but with some variability when phylogenetic uncertainty is taken into account, especially in Anseriformes (Tables 1 and 2). Contrary to our predictions of pattern evolution, bars largely evolved first rather than scales in Galliformes, but in Anseriformes bars or scales evolve from an absence of patterns. However, our analysis demonstrates that spots have evolved from other pre-existing patterns, and therefore, as hypothesized, reaction–diffusion based spatiotemporal differentiation may constrain spots to evolve from an absence of patterning by a minimum of two transitions (Figs. 1 and 3). This occurs in two avian orders that have very different lifestyles, illustrating the importance of development in evolution. Finally, the models over the whole body demonstrate that many mechanisms are conserved from the level of patches, such as spots evolving last, but they also highlight some interesting differences.

There is consistent support for a bias in the direction of plumage evolution at all levels of the analyses, including all patches of plumage with more than two pattern states and the whole body. The main exception to this is a transition from an absence of patterning to spots in the flanks in both orders but only when the phylogeny of all species is used. As flanks are important for signaling, it seems likely that this rare transition is a result of strong selection pressure circumventing developmental constraint. The overall evolutionary trajectory of within-feather patterns suggests that with increasing complexity in the mechanism of pattern formation, different types of patterns become developmentally more accessible. These results are congruent with developmental constraint in this system, but the main pathway, that bars evolve first in Galliformes, does not follow our predictions which may indicate that the developmental basis of scales is more complex than that of bars or there is variation in the mechanism of pattern formation between Anseriformes and Galliformes.

Some support for a relative lack of mutational constraint on bar formation comes from genetic studies. Several independent mutations, both autosomal and Z-linked, can lead to bars from an absence of patterning in birds (chicken: Crawford [27], Muscovy duck: [28]). In the best studied case, the sex-linked barred mutation in chickens, controlled by the *CDKN2A/B* locus, barring is associated with pale bands devoid of melanocytes [29]. A different locus, *ASIP*, controls temporally-related banded patterning in mammalian hairs [30–32], and is a potential candidate for within-feather patterning. Mutations at this locus in quail affect coloration and bar width in individual feathers ([33]; N.I. Mundy and F. Minvielle unpublished data), and *ASIP* expression in developing chicken feathers is spatially variable [34]. Thus the evolutionary origin of bars may be more straightforward than inferred from the reaction diffusion model. Currently, a large gap in our understanding is a plausible mechanism for how these loci might be involved in a reaction–diffusion mechanism.

The whole body model also showed evidence for sequential steps in plumage pattern evolution. If recruitment of patterning mechanisms across local patches were common this would lead to more transitions occurring in the whole body model. However, evidence for this is limited. For Galliformes it is striking that the main features of the within patch models, including a stronger transition from an absence of patterning to bars than scales, occur in the whole body model. For Anseriformes, the picture is mixed – whereas a transition from absence of patterns to scales occurs in the whole body, the transition from absence to bars does not, even though this is strongly supported in the within-patch models. However, taking into account phylogenetic uncertainty there is congruence in bars evolving from an absence of patterns at the level of patches and the global model. Most strikingly, in both orders, spots can only evolve from pre-existing patterns and not from an absence of patterns for the whole body.

We have carried out a broad level analysis of two large sister taxa that are ecologically distinct, and the decision to analyse them separately receives *post hoc* justification from the finding of differences among them. It is of course possible that different transition rates of plumage patterns occur among different clades within these taxa. This is an interesting issue for future investigation. Although we analysed most extant species within Anseriformes and Galliformes (63 % combined), 37 % of species were excluded because of a lack of robust phylogenetic information. The effects of this on our results are unknown, but we note that the absence of these taxa is not different in principle to the absence of an unknowable number of extinct taxa in the dataset. Hybridization is common in both orders: an estimated 41.6 % species of Anseriformes and 21.5 % species of Galliformes hybridize [35]. Hybridization can lead to rapid shifts in phenotype that could cause uncertainty in estimating model transitions.

The effect of categorizing mottled plumage as missing data is unknown. In Anseriformes, there are few species with mottled plumage whereas it is more frequent in Galliformes (Additional file 1: Figure S1, S2). Given that mottled patterns do not appear to have a regular motif, categorizing these patterns is plagued by uncertainties. Having many categories for different types of mottled patterns would likely obscure a signal of a bias in the sequence of plumage pattern evolution, whereas using a single category for a pattern that exists in many states, might overly constrain the model. Therefore, using a category of “unknown” is representative of what is currently known about plumage pattern formation, and using robust Bayesian based analyses based on multi-model inference should largely control for uncertainty [36].

An issue for future consideration is the potential effect of female patterning on the evolution of patterning in males. In both orders studied, there is sexual dimorphism in plumage patterns (Anseriformes: 54 %, Galliformes: 36 %; [37]), which is estrogen-dependent [38, 39]. As a consequence, it was thought that elaborate coloration initially evolved in both sexes via genetic correlation [39–42]. However, currently there is little evidence to suggest that there is genetic correlation in plumage pattern evolution between males and females in Anseriformes and Galliformes [37]. Hence the possibility that certain patterns evolve first in females and are later acquired by males remains, and will be considered in future studies.

## Conclusions

Similar plumage patterns have evolved in many distantly and closely related species of birds [9, 11, 43]. We demonstrated that plumage pattern evolution follows sequential steps that is congruent with developmental constraint. Overall there was remarkable similarity in the trajectories of pattern evolution in Galliformes and Anseriformes, suggesting that the constraint is similar in the two orders, despite large ecological differences. As suggested by Price and Pavelka [16] the role of natural selection may be “fine-tuning the appearance of the pattern, fixing and maintaining pattern elements at a given level of expression, and modifying behavioral and other features to maximize the patterns’ utility” on the basis of the order that patterns evolve. We suggest that the sequential nature of plumage pattern evolution may be caused by the underlying dynamics of the developmental system of patterning, which may be of general significance to birds. Our study highlights the possibility of testing hypothetical models of development with comparative methods as a complement to experimental studies.

## Methods

### Phylogenies

We searched the literature for published species level relationships. The best available phylogenies on the basis of species coverage, inclusion of mtDNA and where possible nuclear DNA, and inclusion of branch length information, are as follows: Anseriformes (mtDNA only), 118 spp. (73 %) - [44]; Galliformes, 170 spp. (59 %) - [10]. These phylogenies are derived from Maximum Likelihood and/or Bayesian inference and together cover all families and 63 % of extant species across the two orders [45]. Each analysis was conducted in two ways to maximise taxonomic coverage and account for phylogenetic uncertainty: first, we used the consensus tree representing all species per phylogeny. Second, to examine the robustness of our results, we collapsed branches with low Bayesian probability (<=0.95) into twigs (Additional file 1: Figure S1-S2). In Anseriformes, this resulted in 33 species being collapsed into four twigs (*see* Additional file 1 for further detail). In Galliformes, collapsing low probability branches resulted in 78 species being collapsed into twelve twigs.

We collected plumage pattern information from each species (nominate subspecies where applicable) represented in these phylogenies from museum skins at the Natural History Museum at Tring and the University Museum of Zoology, Cambridge.

### Data collection and coding

Current developmental evidence suggests that the default plumage phenotype in males and females in Anseriformes and Galliformes is the male plumage, in the sense that estrogen is required to produce female plumage but that testosterone is not required to make male plumage [38]. Therefore, we collected plumage pattern information for the seven patches of plumage over the body for the males of each sample species (Fig. 2). We assigned the character state of each of the seven feather patches as scales, bars, spots, or an absence of patterns, following the description by Prum and Williamson [13]. Variation in scoring plumage patterns between ten ornithologists is modest [43]. In the study reported here all plumage patterns were scored by T-LG. Some species exhibit what appear to be longitudinal stripes along feathers, but on closer inspection are an angular version of scales with a central pigment patch, and were scored as scales, e.g. the breast and nape plumage of the vulturine guineafowl (*Acryllium vulturinum*) (Fig.1a*in* [13]). A small number of species in this study have chevron patterns – Anseriformes – 2 *spp.*, Galliformes – 5 *spp.*. Given that chevrons are rare in these orders and that they are similar to patterns made of bars, in that the borders do not meet to create a central pigment patch, we scored chevrons as bars (Fig. 1e and 6e *in* [13]).

For most species sampled, the type of within-feather patterning across the vane of each feather, as well as between individuals of the same species, was the same for each patch considered. However, in a rare number of cases there was variation between individuals. To focus on the most developmentally relevant patterns in these rare cases we recorded the pattern that covered the majority of the feathers, in the patch under consideration, and where relevant, the predominant pattern in the majority of individuals sampled. For example, in the Natal francolin (*Pternistes francolinus*), the feathers in the flanks can have both bars and scales (Fig. 2). In the example depicted, bars cover the majority of the feathers in the flanks, and this individual would have been assigned as having bars. However, in most individuals of the sample population of the Natal francolin, scales predominantly covered most of the feathers in the flanks, and were considered representative of this species.

An additional type of pattern, mottled plumage, is present in many birds. It is currently unknown whether all mottled patterns can be considered homologous, or whether they may be classified into discrete types based on the size, shape and distribution of pigmentation across the vane of the feather. Therefore, mottled plumage was scored as unknown. In the tail of Anseriformes, only one type of plumage pattern has evolved which is only exhibited in 7 species that are in derived clades (Additional file 1: Figure S1). Therefore, we removed the tail patch from the analysis.

In the analyses where branch lengths with a low probability were removed, the twigs were coded as having all of the pattern states of each species represented in the removed branch. For example, if the species in the branch that was collapsed into Twig X had spots and bars, Twig X was scored as having both pattern types.

To investigate whether within-feather pattern evolution in one patch of plumage may precede and/or promote evolution of patterning in other patches, we conducted further analyses over the whole body (Fig. 2). Where a species has multiple types of plumage patterns over the body, the pattern that had evolved most recently across all patches (as indicated by the summary model of local evolution) was considered representative. For example, if from an absence of patterns evolved bars followed by spots, species that contained both bars and spots were coded as having spots for analyses of the whole body model. In Anseriformes, in the summary model of local evolution within patches there is no conflict in the order of transitions and the order of evolution is clear in models derived from the all species phylogeny. However, in the robust analysis scales or bars could have evolved first. In Galliformes, bars evolve from an absence of patterns and the next pattern to evolve from bars could be either scales or spots in both the unmodified and modified phylogenies (*see* Results).

We took this uncertainty into account by examining each possible trajectory for comparison. For example, males of the satyr tragopan (*Tragopan satyra*) have scales on the flanks and vent, and spots on the breast. In the analysis of the whole body in Galliformes, where plumage patterns over the whole body is collapsed into one character, we compared whether assigning either scales or spots as the character that had evolved last created conflict in the analysis. Similarly, in the robust analysis for Anseriformes, we compared whether assigning either scales or bars as the character to evolve first created conflict in the analysis.

### Modeling of plumage pattern evolution

We modeled plumage pattern evolution over the phylogenies of each order to estimate the evolutionary transitions between patterns, allowing us to derive a model of the probable evolutionary pathways between plumage pattern phenotypes. Anseriformes and Galliformes live in different habitats, which may alter the evolutionary trajectory of each order [25, 26]. Therefore, we examined each order separately to assess for similarity and differences in their evolutionary history. To estimate plumage pattern evolution in each order, we used the Reversible Jump Markov Chain Monte Carlo Multistate option in BayesTraits v.2 [36, 46].

Markov Chain Monte Carlo (MCMC) is based on the proposition that traits can repeatedly evolve between any possible state on any branch of the tree. To estimate the rate of change between states, the Markov chain samples the plumage patterns at the internal nodes of the tree, in proportion to their probability, which is conditioned on the values at the tips. The rate of change between states was allowed to vary over each transition. New rate parameter values are proposed in successive steps in the Markov chain resulting in a posterior sample distribution of rate coefficients and ancestral states. The rate coefficients of each model of pattern evolution is visited in direct proportion to its posterior probability in the sample distribution [36]. Given that there are four pattern states, which in turn offer many parameters that describe evolution between plumage patterns, we used Reversible Jump MCMC (RJMCMC).

RJMCMC integrates rate restrictions by searching the posterior distributions of model parameters to avoid over parameterization. As such, we allowed BayesTraits to propose transition rates of plumage pattern evolution without restriction (e.g. we did not constrain any rate parameters to equal 0 based on *a priori* predictions) thereby making the analysis conditional on the data rather than our hypothesis [42]. For example, we hypothesized that the greatest number of steps are required to evolve spots as a consequence of having the strictest developmental parameters and therefore do not evolve directly from an absence of patterns (Fig. 1). In transition rate models that support this hypothesis, a rate parameter between an absence of patterns and spots equals 0, and therefore does not occur. This allows both incremental and non-sequential changes to occur in any direction and avoids imposing potentially false hypothesis based predictions.

Potential models of plumage pattern evolution visited by the Markov chain are distinct from the most probable model of plumage pattern evolution. The former describes the proposed models of plumage pattern evolution that make up the posterior sample distribution, whereas the latter is derived from statistically evaluating the posterior sample distribution. Each model of plumage pattern evolution is composed of a unique combination of transition rate parameters with values fixed to zero or are sampled as free parameters with positive values. Rate parameters fixed to zero were interpreted as an evolutionary transition that does not occur, and free rate parameters with a positive value were interpreted as evidence for an evolutionary transition that does occur. Therefore, qualitatively, each unique model of plumage pattern evolution is composed of transitions that do not occur, and transitions that do occur.

Null model testing and model comparisons were conducted by assessing the posterior distribution of unconstrained models. If there were no developmental constraint, such as where natural selection drives plumage patterns to evolve in any direction, forward and backward evolutionary transitions between all pattern states would occur – the full (null) model (Fig. 1). Therefore, if plumage pattern evolution is random, the full model would be visited more frequently than expected by chance. Conversely, if sequential or non-sequential evolutionary transitions were more probable, then models with these transitions would be most probable. In assessing the models of evolution without constraining any transitions, each unique model of pattern evolution is compared with every other possible model of pattern evolution (statistical methods are described in the next section).

In BayesTraits we modeled the rates of plumage pattern evolution using a hyperprior with a gamma distribution defined by an empirical Bayes estimator [36]. For each analysis, we discarded the burn-in. The Markov chain was allowed to run for an infinite number of iterations and was terminated when convergence was reach across four independent runs (<1 lnHM). The number of chains required to reach convergence varied between patches of plumage (Additional file 1: Table S1). After convergence was reached, we checked the posterior sample distribution for autocorrelation. Where autocorrelation was present, the posterior sample distribution was reduced. The average rate of transition for each patch of plumage and over the whole body, are presented in the online appendices.

### Model priors and modeling parameters

The prior density on the free transition rate parameters were estimated using an empirical Bayes estimator (where the interval of the hyperprior is defined by the average and standard deviation of the maximum likelihood of all rate parameters) to reduce bias and uncertainty in choice of priors [36]. We used a hyperprior approach with a gamma distribution as our empirical Bayes estimator values had an intermediate range. The intervals were estimated for each analysis, for each patch of plumage or the whole body, in each group separately. For the analysis of independent evolution within patches of plumage, in each phylogeny, we sampled every 100,000th generation (7 × 2 = 14 individual analyses). The first 500,000 generations of RJMCMC (burn-in) were discarded to ensure parameter space was sufficiently explored.

Each analysis of separate plumage patches and the global model, per order, was run four times to ensure convergence had been reached within analyses as indicated by a stable harmonic mean that varied by <1 lnHM across all four runs. We checked for autocorrelation using the Box-Ljung test statistic in SPSS v22 at lag 1 (IBM Corp.). A Ljung-box *P* > 0.05 was interpreted as indicating no autocorrelation (Additional file 1: Table S1). There was autocorrelation in four models (all species - Anseriformes: wing and rump, Gallifromes: belly; robust – Anseriformes: rump) and we thinned the posterior sample distribution of these models of plumage pattern evolution to every 10th iteration (1,000,000th model), preserving the order in which the models were visited. This resulted in a posterior sample distribution of ~20,000-53,000 per model.

### Statistical analysis

The most probable models of plumage pattern evolution, each with their own most probable ancestral state of patterning, are visited in proportion to their Bayesian posterior probability. Given that there are four states of patterning in this analysis, each model has twelve possible evolutionary transitions. Each transition between each type of pattern can have a transition rate that is above zero (occurs) or a rate of zero (does not occur). To account for the effect of varying numbers of zero and non-zero transitions on the probability of each model of pattern evolution we calculated the prior odds of each model using binomial numbers for transitions that do *not* occur, and bell numbers for transitions that occur, combined (Additional file 1: Table S1 & S2**;***see* [47] for a detailed explanation of calculations used). The posterior odds were derived from the posterior sample distribution, and compared with the prior odds using Bayes Factors.

There can be multiple probable models of evolution in the top model set. As a consequence there is uncertainty in the most probable model of plumage pattern evolution. Therefore, we treated the analysis of the posterior sample distribution of models in a multiple-model framework using multimodel inference [37, 48, 49]. Similar approaches are used in multimodel inference using AIC. However, AIC is not philosophically equivalent to Bayesian modelling. Instead we used BayesFactors to rank our competing models. To derive a top model set we used a threshold of a BayesFactor of > =2, which is considered positive evidence [48, 50].

The ancestral state of plumage as well as rate parameters of each unique model of pattern evolution can vary widely in whether they are fixed to zero, or sampled as free parameters with positive values. Therefore, we calculated the marginal probability (MP) per ancestral pattern and of each transition parameter occurring, or not occurring. To account for uncertainty, we calculated MP from the entire posterior sample distribution. For example, the MP of each type of pattern being the ancestral state in the top model set = the number of models in which this pattern is ancestral/n total models. For each individual transition parameter in each unique model of plumage pattern evolution in the top model set MP = the number of models in which this transition parameter occurs/n total models, and MP = the number of models in which this transition parameter does not occur/n total models.

**Fig. 5:**
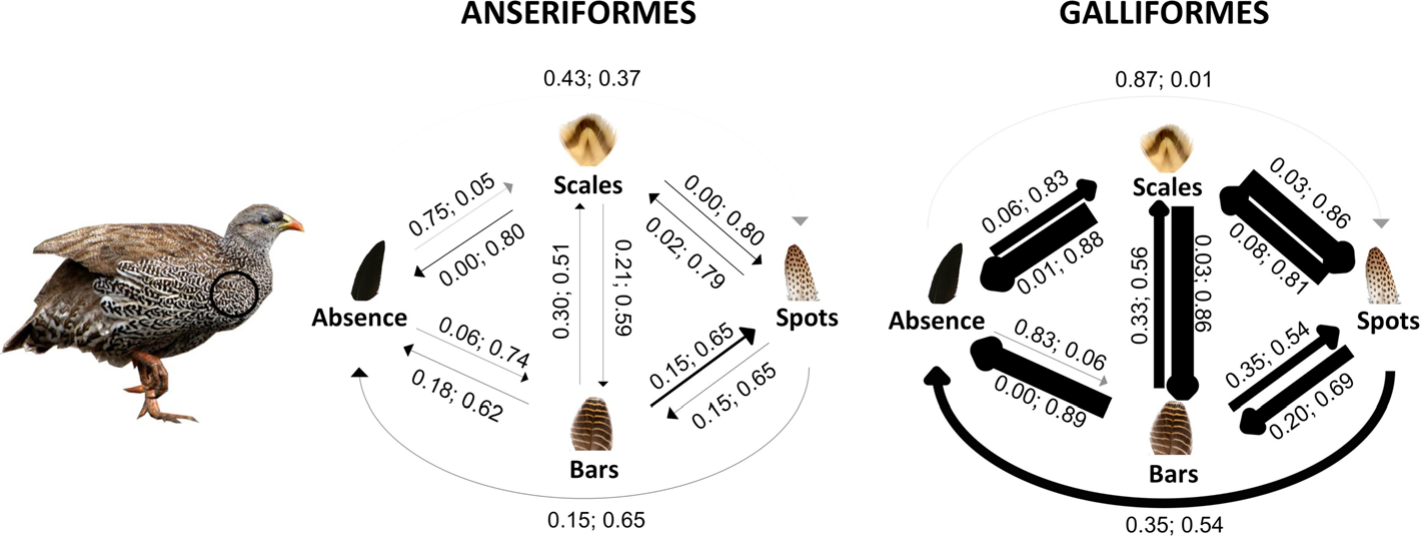
The marginal probability of evolutionary transitions between plumage patterns in the feather tract of the breast in Anseriformes and Galliformes using unmodified phylogenies. Next to each transition is the marginal probability of a transition not occurring, followed by the marginal probability of it occurring. The width of the transition line is proportional to the rate of transition. Where the probability of a transition occurring is less than the probability of the transition not occurring, the transition line is grey indicating that it most probably does not occur. Conversely, where the marginal probability of it occurring is higher than not occurring, the transition line is black indicating that the transition probably occurs. The marginal probability of occurring and not occurring does not equal due to variation in the transitions represented in the top model set

The final marginal probability was calculated by cumulatively adding the MP of each model in the top model set for each ancestral state of patterning and for each evolutionary transition where it does *not* occur, as well as where it occurs, for comparison [46]. For example, in the breast of the galliform birds, the marginal probability (MP) of an absence of patterning *not* being the most probable ancestral state is 0.00 in the top model set whereas the MP of an absence of patterning *being* the most probable ancestral state is 0.89 (e.g. Table 1). In addition, the MP of scales, bars and spots *not* being the most probable ancestral state is 0.89 versus 0.00 of *being* the ancestral state. Assessing a transition from an absence of patterns to spots, the MP of the transition rate parameter describing it as *not* occurring is 0.87 and its MP of being non-zero is 0.01 (Fig. 5). Together this shows that an absence of patterns is most probably the ancestral state in the breast of galliform birds, and a transition from an absence of patterns to spots most probably does *not* occur. The MP in the top model set accounts for variation in the entire posterior sample distribution, therefore the sum of the MP of a transition *not* occurring and occurring rarely equals 1 as this requires every model in the posterior sample distribution to have the same result for that transition.

## Additional file

Additional file 1:
**Figure S1a.** The distribution of plumage pattern traits per patch of plumage in Anseriformes. Empty traits indicate that the type of pattern is unknown and/or is mottled plumage. Branches that were collapsed into twigs due to low branch probability are indicated with a red line (*see* Supplementary Methods above). Each type of plumage pattern is found in extant species and although there is some variation in the most probable ancestral state, where there is support it is for an absence of patterns. **Figure S1b.** Distribution of plumage pattern traits per patch of plumage in Galliformes. Branches that were collapsed into twigs due to low branch probability are indicated with a red line (*see* Supplementary Methods above). Empty traits indicate that the type of pattern is unknown and/or is mottled plumage. Each type of plumage pattern is found in extant species and the most probable ancestral state of plumage is an absence of patterns. **Figure S2a.** Local plumage pattern evolution within individual patches, in Anseriformes and Galliformes using unmodified trees. The width of each evolutionary step is proportional to the average rate per model. Beside each evolutionary step is the marginal probability of each transition not occurring, followed by the marginal probability of it occurring. Where the transition probably does not occur, the transition line is grey. Conversely, where the transition probably does occur, the transition line is black. Equivocal transitions, where the marginal probability is = < 0.05 difference between not occurring and occurring, are indicated by a grey dashed line. **Figure S2b.** Plumage pattern evolution over the whole body, in Anseriformes and Galliformes using unmodified trees. To examine the effects of uncertainty in the order of plumage pattern evolution in Galliformes we modeled the effect of scales or spots being more derived. The width of each evolutionary step is proportional to the average rate in the top model set. Beside each evolutionary step is the marginal probability of each transition not occurring, followed by the marginal probability of it occurring. Where the transition probably does not occur, the transition line is grey. Conversely, where the transition probably does occur, the transition line is black. Equivocal transitions, where the marginal probability is = < 0.05 difference between not occurring and occurring, are indicated by a grey dashed line. **Figure S3a.** Local plumage pattern evolution within individual patches, in Anseriformes and Galliformes using trees that only have branches with a high Bayesian probability. The width of each evolutionary step is proportional to the average rate per model. Beside each evolutionary step is the marginal probability of each transition not occurring, followed by the marginal probability of it occurring. Where the transition probably does not occur, the transition line is grey. Conversely, where the transition probably does occur, the transition line is black. Equivocal transitions, where the marginal probability is = < 0.05 difference between not occurring and occurring, are indicated by a grey dashed line. **Figure S3b.** Plumage pattern evolution over the whole body, in Anseriformes and Galliformes trees that only have branches with a high Bayesian probability. To examine the effects of uncertainty in the order of plumage pattern evolution in Galliformes we modeled the effect of scales or spots being more derived. The width of each evolutionary step is proportional to the average rate in the top model set. Beside each evolutionary step is the marginal probability of each transition not occurring, followed by the marginal probability of it occurring. Where the transition probably does not occur, the transition line is grey. Conversely, where the transition probably does occur, the transition line is black. Equivocal transitions, where the marginal probability is = < 0.05 difference between not occurring and occurring, are indicated by a grey dashed line. **Table S1.** Prior probability of encountering models of n parameters calculated from binomial for Z (where Z = n parameters that are set to 0 i.e. do not occur) and Bell numbers for models with 12 possible transition rates. **Table S2.** Prior probability of encountering models of n parameters calculated from binomial for Z (where Z = n parameters that are set to 0 i.e. do not occur) and Bell numbers for models with three pattern states encompassing 6 possible transitions. (DOCX 12836 kb)
