# Supplementary material for: Evolutionary pathways to convergence in plumage patterns
Source: BMC Evol Biol. 2016 Aug 31;16(1):172. doi: 10.1186/s12862-016-0741-x (PMC5006497; doi:10.1186/s12862-016-0741-x)
Supplement: Additional file 1: — Figure S1a. The distribution of plumage pattern traits per patch of plumage in Anseriformes. Empty traits indicate that the type of pattern is unknown and/or is mottled plumage. Branches that were collapsed into twigs due to low branch probability are indicated with a red line (see Supplementary Methods above). Each type of plumage pattern is found in extant species and although there is some variation in the most probable ancestral state, where there is support it is for an absence of patterns. Figure S1b. Distribution of plumage pattern traits per patch of plumage in Galliformes. Branches that were collapsed into twigs due to low branch probability are indicated with a red line (see Supplementary Methods above). Empty traits indicate that the type of pattern is unknown and/or is mottled plumage. Each type of plumage pattern is found in extant species and the most probable ancestral state of plumage is an absence of patterns. Figure S2a. Local plumage pattern evolution within individual patches, in Anseriformes and Galliformes using unmodified trees. The width of each evolutionary step is proportional to the average rate per model. Beside each evolutionary step is the marginal probability of each transition not occurring, followed by the marginal probability of it occurring. Where the transition probably does not occur, the transition line is grey. Conversely, where the transition probably does occur, the transition line is black. Equivocal transitions, where the marginal probability is = < 0.05 difference between not occurring and occurring, are indicated by a grey dashed line. Figure S2b. Plumage pattern evolution over the whole body, in Anseriformes and Galliformes using unmodified trees. To examine the effects of uncertainty in the order of plumage pattern evolution in Galliformes we modeled the effect of scales or spots being more derived. The width of each evolutionary step is proportional to the average rate in the top model set. Beside each evolutionary ste [file 12862_2016_741_MOESM1_ESM.docx]

**Online appendices**

Evolutionary pathways to convergence in plumage patterns

T-L Gluckman and N.I. Mundy

**Supplementary methods**

In the phylogenies utilized in this analysis, some branch lengths had a low Bayesian probability [1,2]. To examine the effect of this on our analyses, branches with a probability of <0.95 were collapsed into twigs representing the pruned branches.

In Anseriformes, 33 species were collapsed into four twigs (Fig. S1a): Twig 1 – *Anas clypeata, A. rhynchotis, A. smithii, A. c. cyanoptera, A. discors, A. c. septentrionalium, A. platalea, A. v. puna, A. v. versicolor, A. hottentoti, A. querquedula, A. formosa, Amazonetta brasiliensis, Speculanas specularis, Lophonetta specularioides, Tachyeres pteneres;* Twig 2 – *Tadorna ferruginea, T. tadornoides, T. cana, T. tadorna, Alopochen aegyptiacus;* Twig 3 – *Anser albifrons, A. erythropus, A. fabalis, A. anser, A. cygnoides, A. brachyrhynchus, A. indicus, A. rossi;* Twig 4 – *Branta Canadensis, B. sandvicensis, B. leucopsis, B. ruficollis*.

In Galliformes, 78 species were collapsed into 12 twigs (Fig. S1b): Twig 1 – *Megapodius cumingii*, *M. tenimberensis;* Twig 2 – *M. decollatus, M. forstenii, M. freycinet, M. eremita, M. reinwardt;* Twig 3 – *M. layardi, M. pritchardii*; Twig 4 – *Arborophila gingica, A. rufogularis, A. rufipectus, A. torqueola, A. javanica, Rollulus roulroul,* Twig 5 – *Centrocercus urophasianus, Dendragapus obscurus, Tympanuchus cupido, T. phasianellus, T. pallidicinctus,* Twig 6 – *Dendragapus falcipennis, Falcipennis canadensis, Tetrao mlokosiewiczi, T. tetrix, T. parvirostris, T. urogallus;* Twig 7 – *Lagopus lagopus, L. mutus, L. leucurus;* Twig 8 – *Crossoptilon crossoptilon, C. harmani, C. drouyni;* Twig 9 – *Syrmaticus ellioti, S. humiae, S. Mikado, S. soemmerringii;* Twig 10 – *Bambusicola fytchii, B. thoracica, Gallus gallus, G. lafayetti, G. varius, G. sonnerati, Peliperdix coqui, Scleroptila africanus, S. levaillantii, S. shelleyi, S. levalliantoides, S. finschi, Dendroperdix sephaena, Francolinus pondicerianus, F. francolinus, F. lathami;* Twig 11 – *Alectoris Barbara, A. chukar, A. rufa, A. magna, A. philbyi, A. graeca, A. melanocephala, Tetraogallus himalayensis, T. tibetanus, Perdicula asiatica, P. adspersus, P. capensis, P. natalensis, P. griseostriatus, P. afer, P. leucoscepus, P. swainsonii, P. squamatus, P. castaneicollis, C. chinensis, C. ypsilophora, C. coturnix, C. japonica, Francolinus pintadeanus;* Twig 12 – *Polyplectron bicalcaratum, P. chalcurum.*


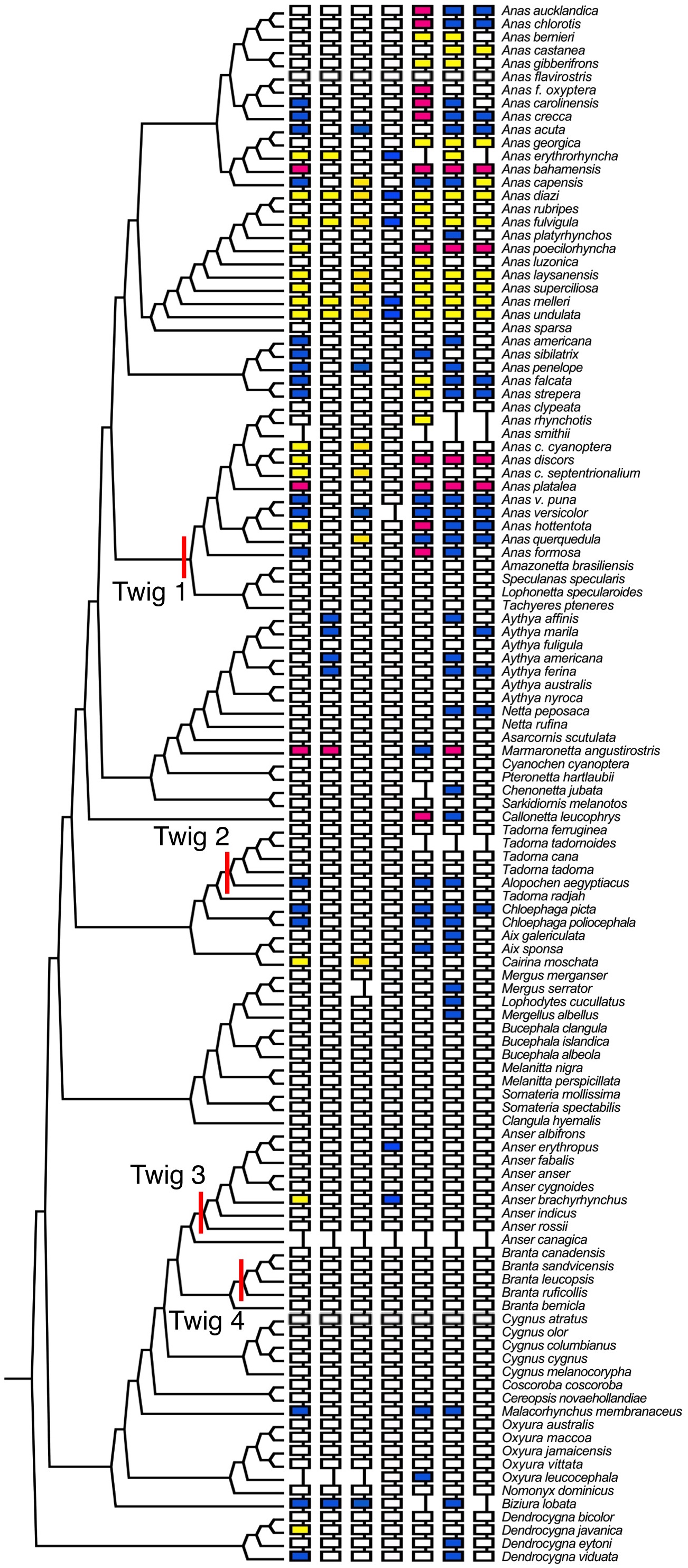


**Nape**

**Rump**

**Tail**

**Breast**

**Flank**

**Vent**

**Wing**


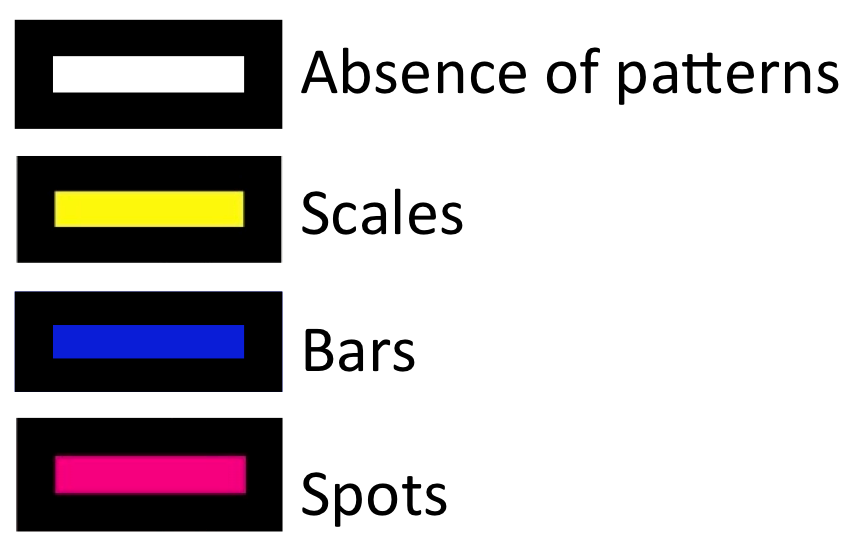


Fig. S1a. The distribution of plumage pattern traits per patch of plumage in Anseriformes. Empty traits indicate that the type of pattern is unknown and/or is mottled plumage. Branches that were collapsed into twigs due to low branch probability are indicated with a red line (*see* Supplementary Methods above). Each type of plumage pattern is found in extant species and although there is some variation in the most probable ancestral state, where there is support it is for an absence of patterns.


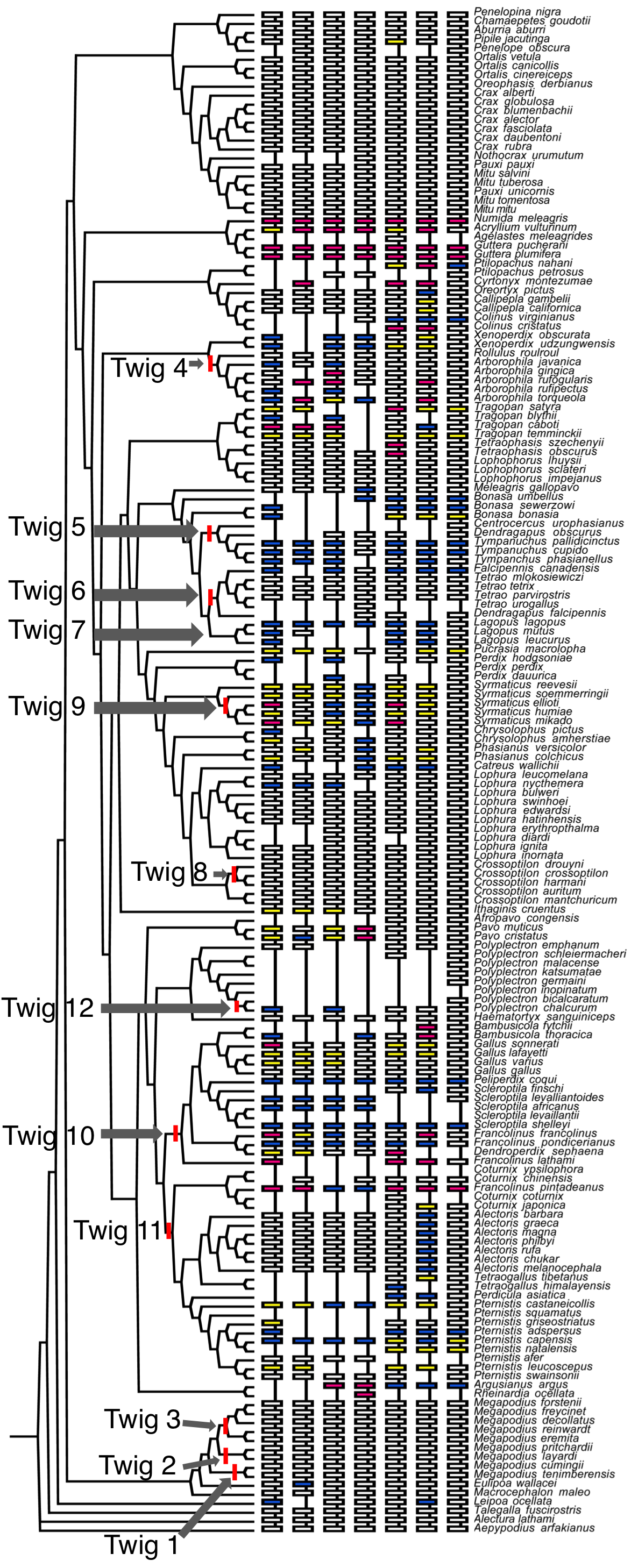


**Vent**

**Nape**

**Wing**

**Rump**

**Tail**

**Breast**

**Flank**


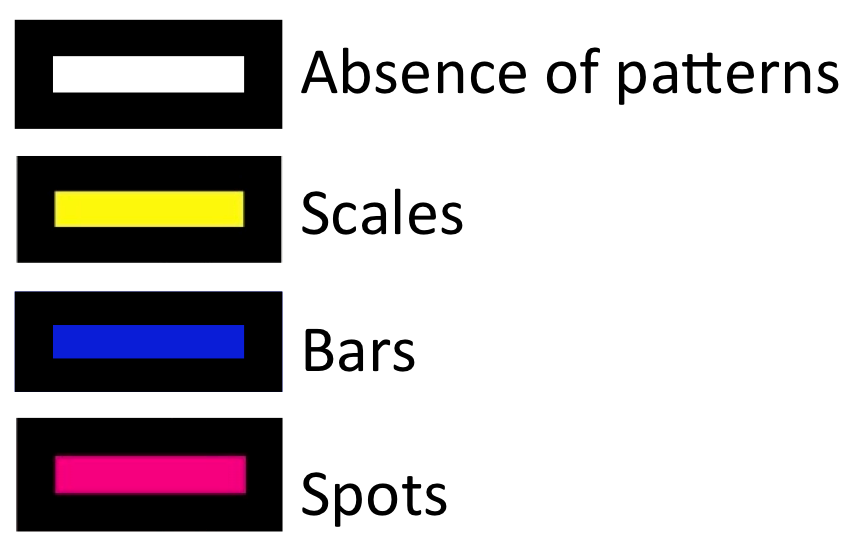


Fig. S1b. Distribution of plumage pattern traits per patch of plumage in Galliformes. Branches that were collapsed into twigs due to low branch probability are indicated with a red line (*see* Supplementary Methods above). Empty traits indicate that the type of pattern is unknown and/or is mottled plumage. Each type of plumage pattern is found in extant species and the most probable ancestral state of plumage is an absence of patterns.

**ANSERIFORMES GALLIFORMES**

UPPERPARTS


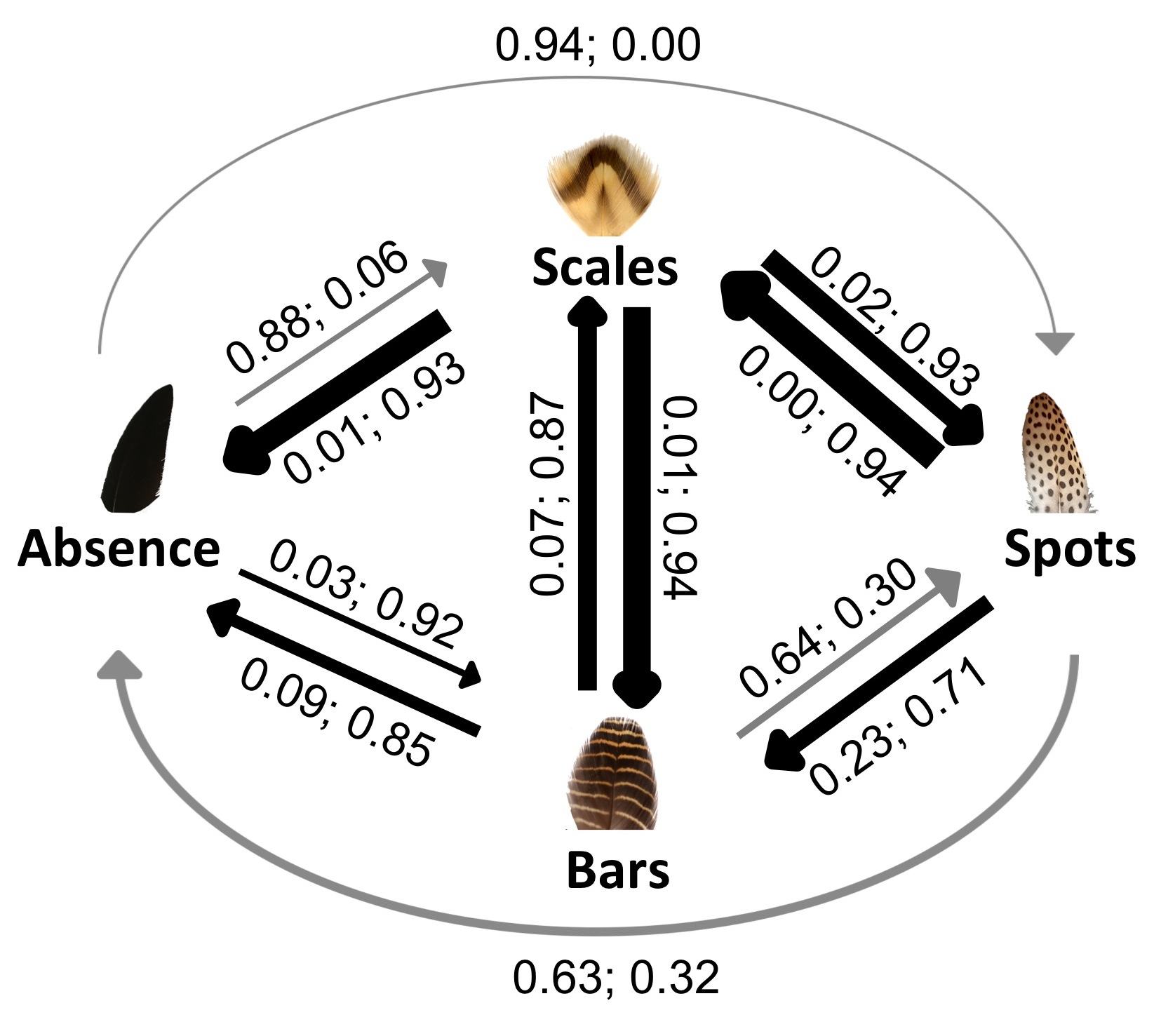

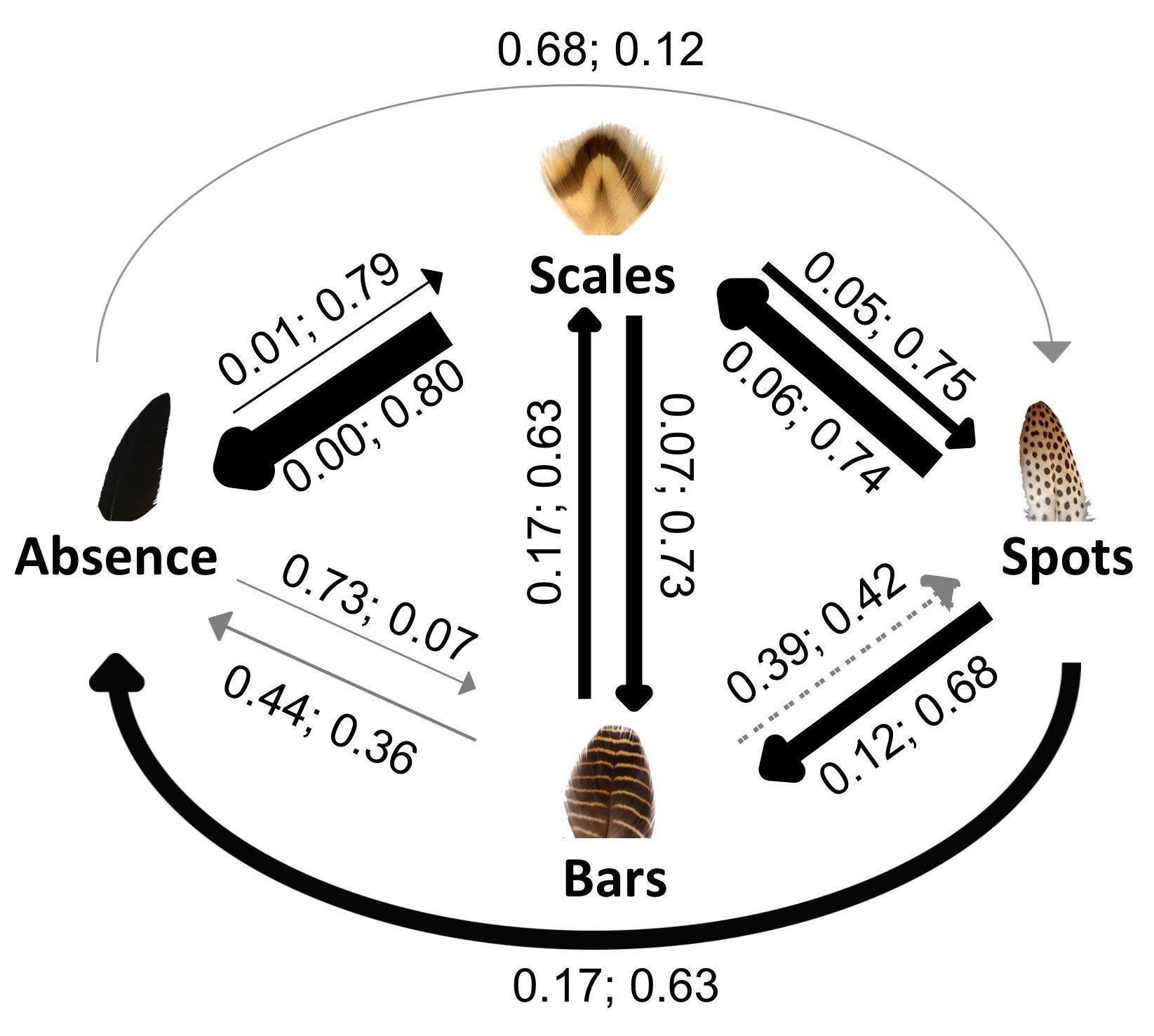
Nape


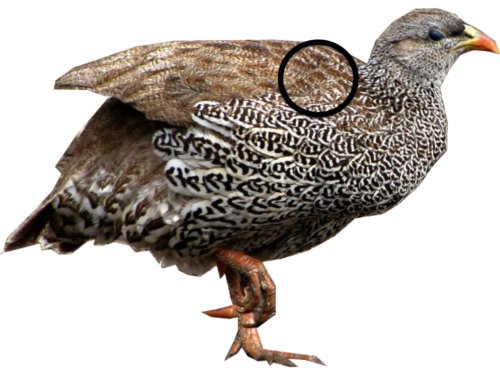


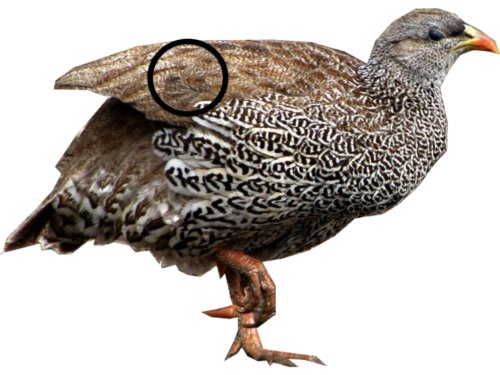

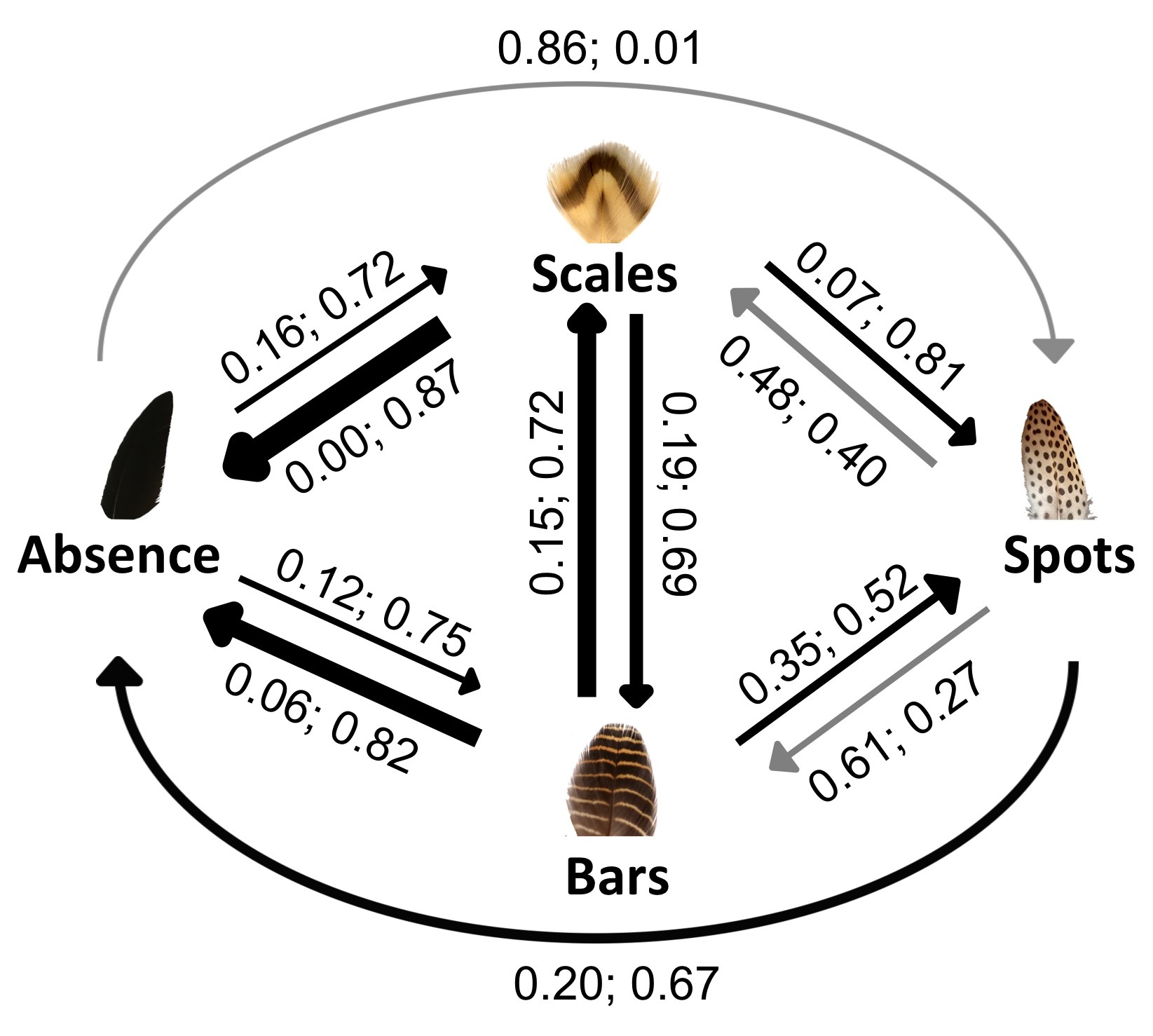
Wing


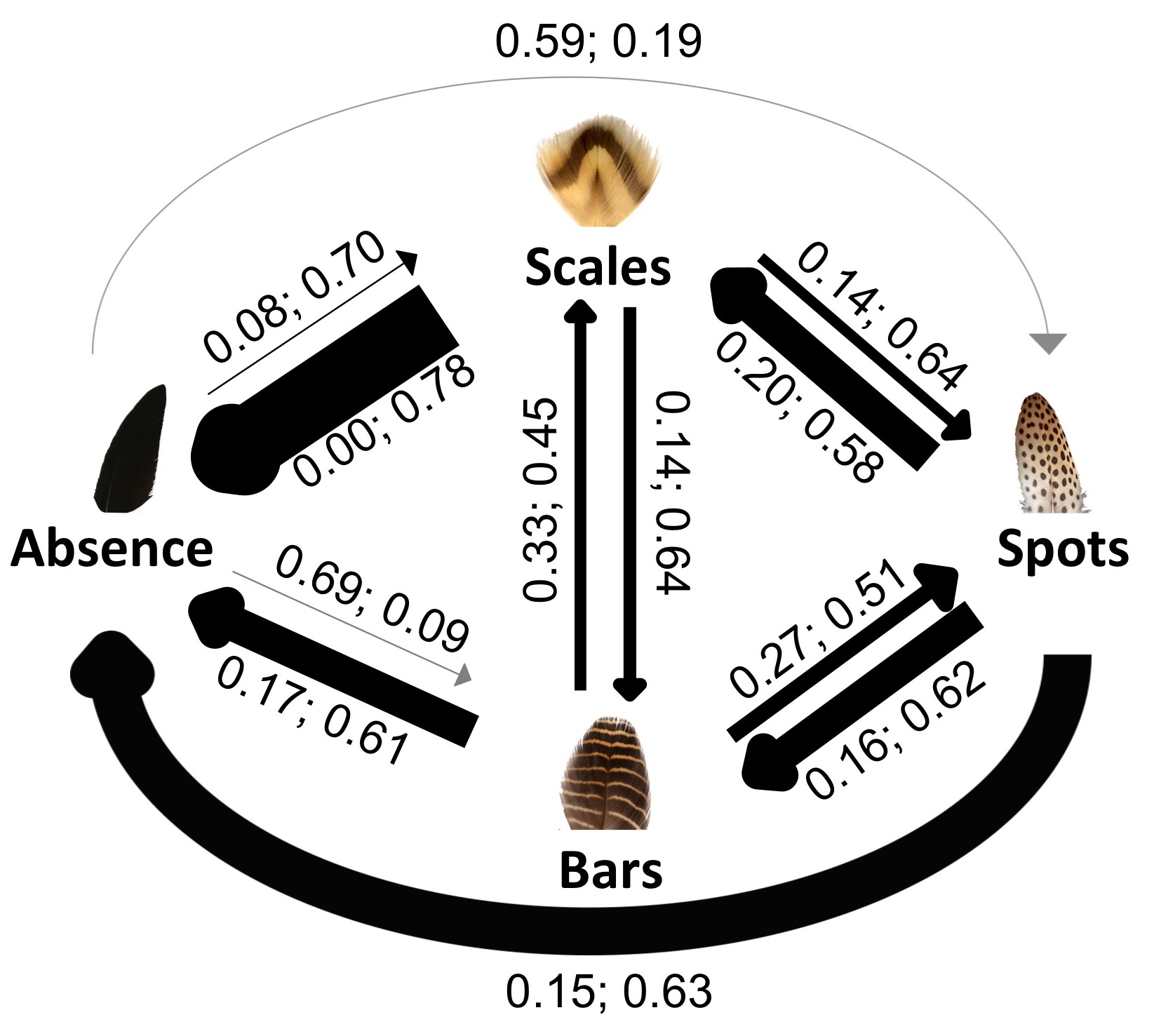


Rump


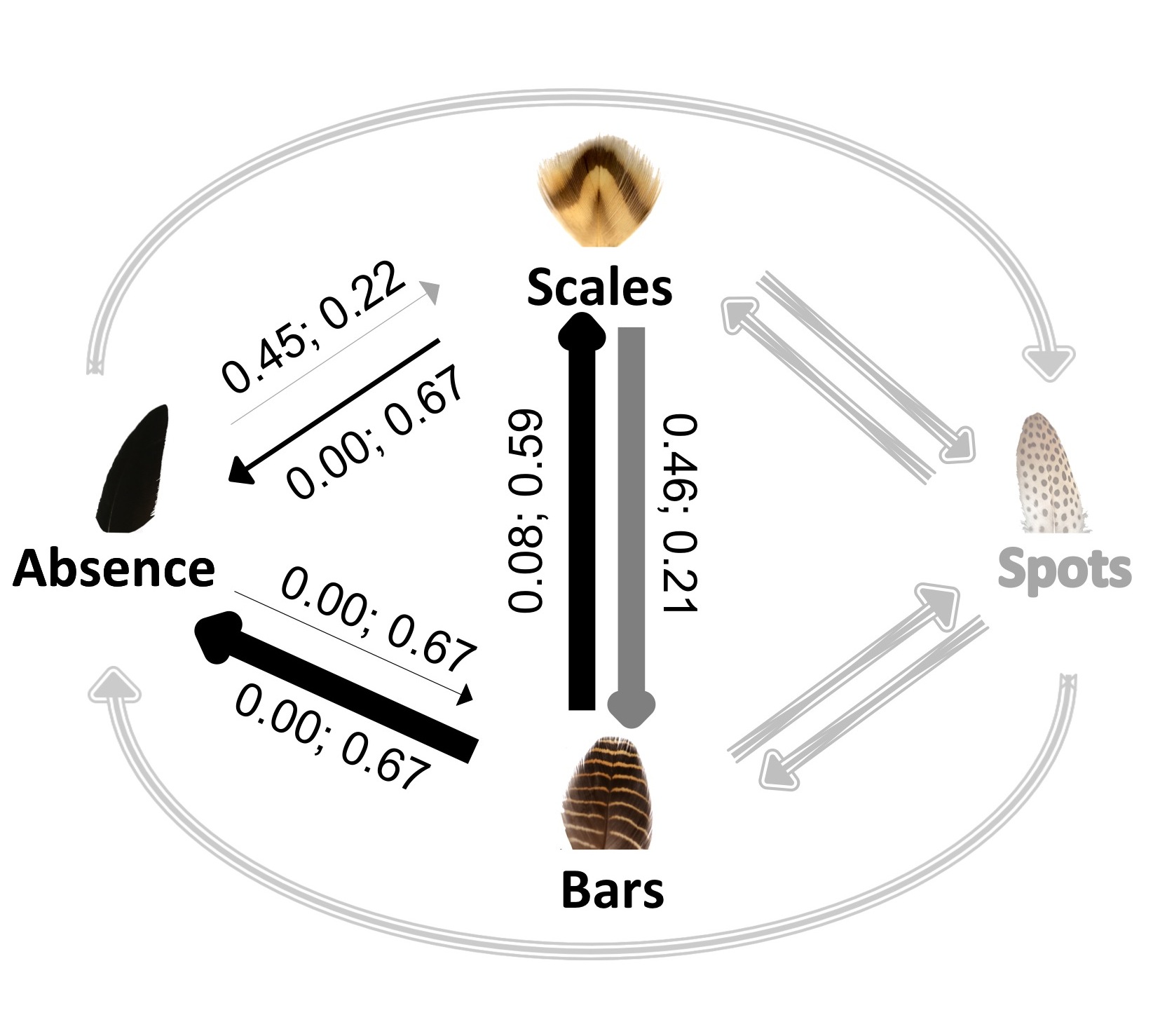

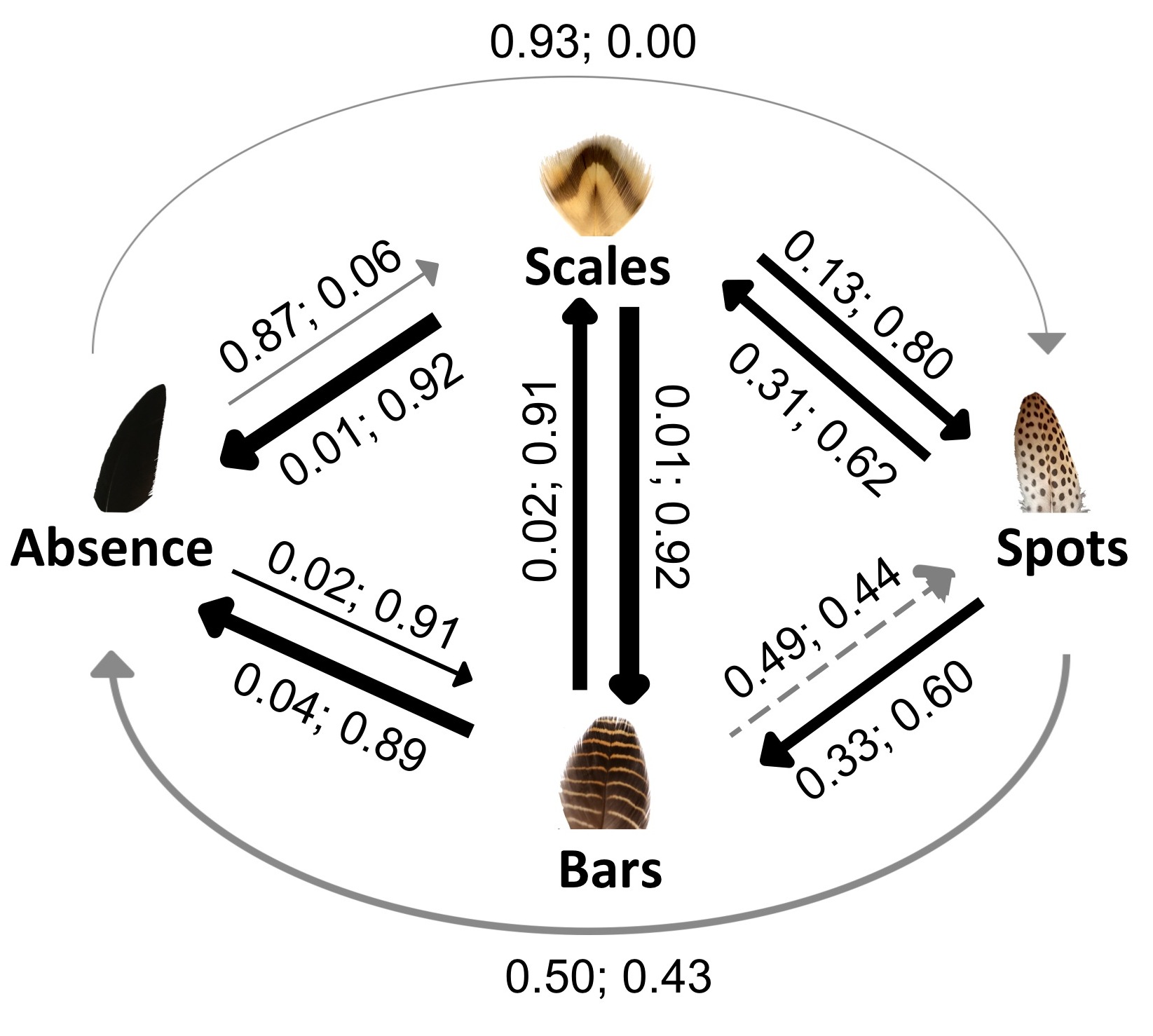

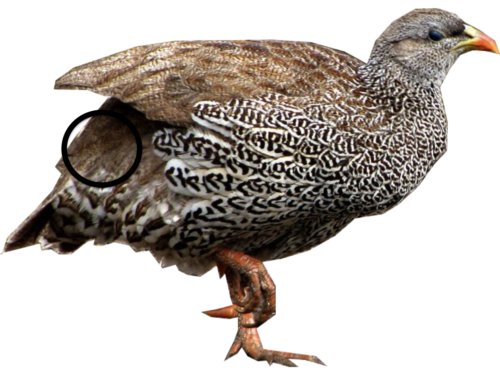


Tail


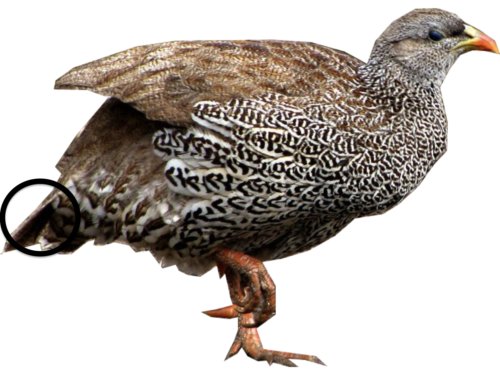


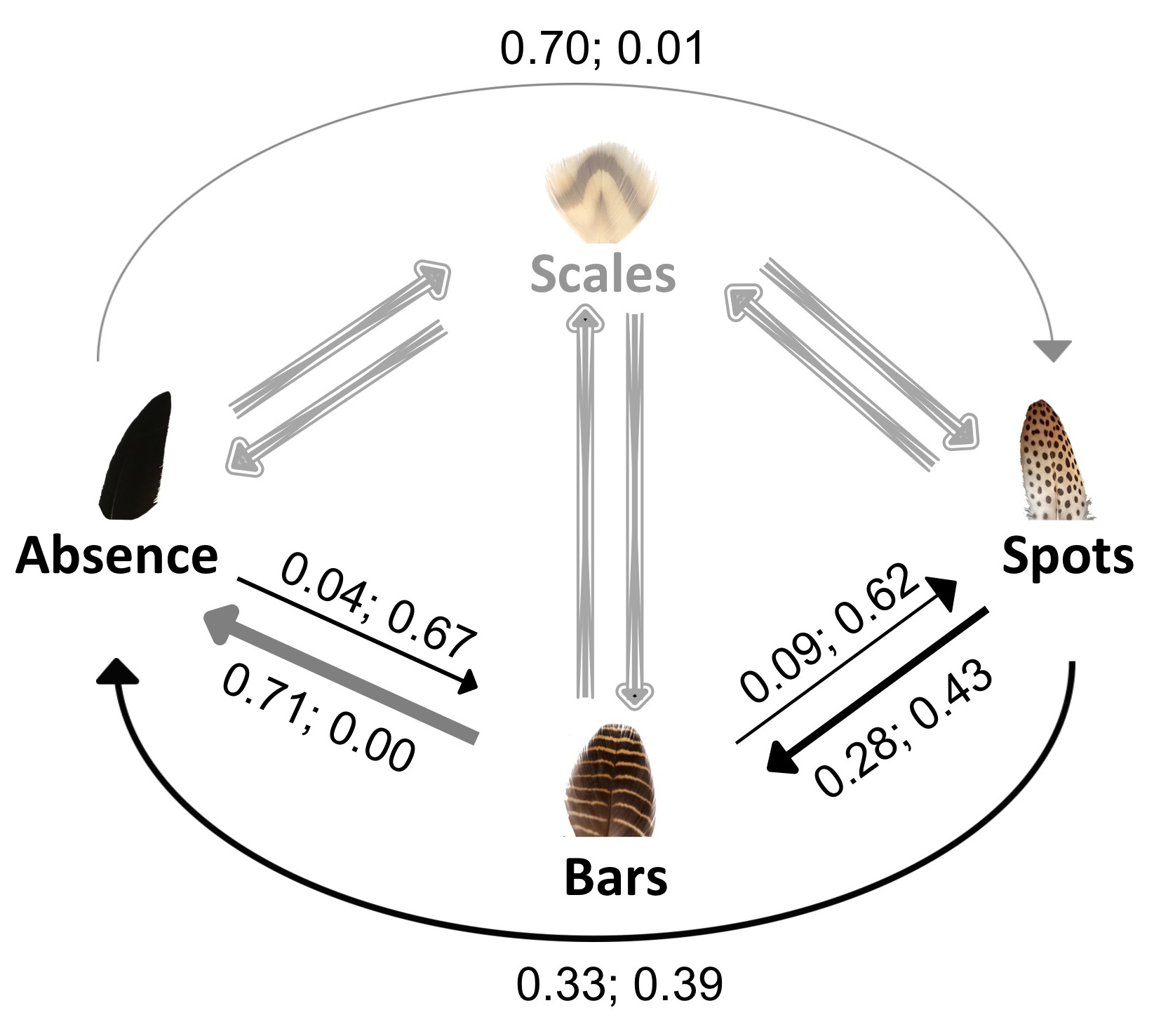


UNDERPARTS


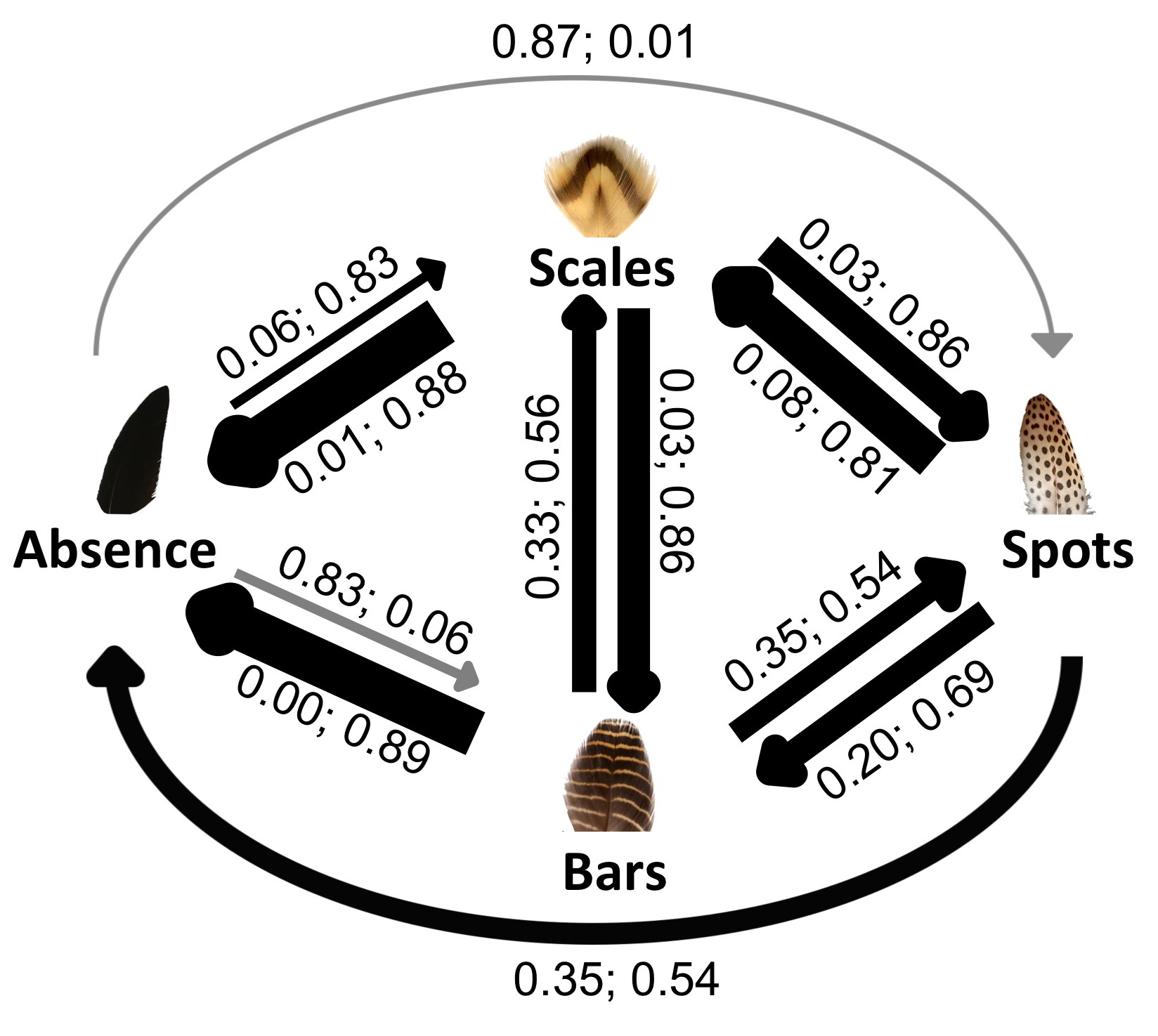
Breast


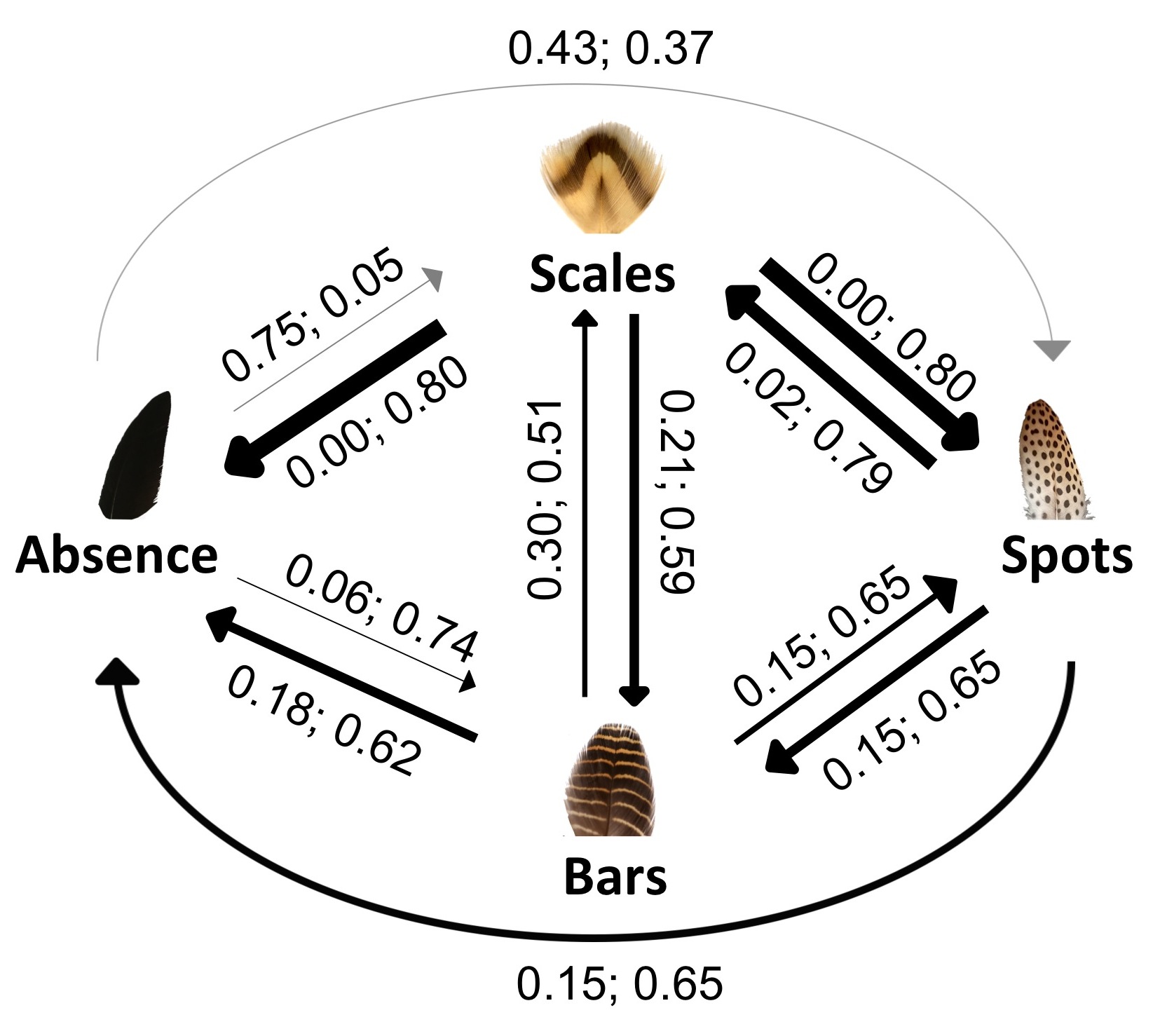

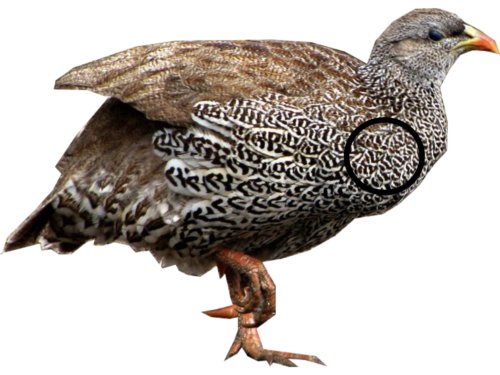


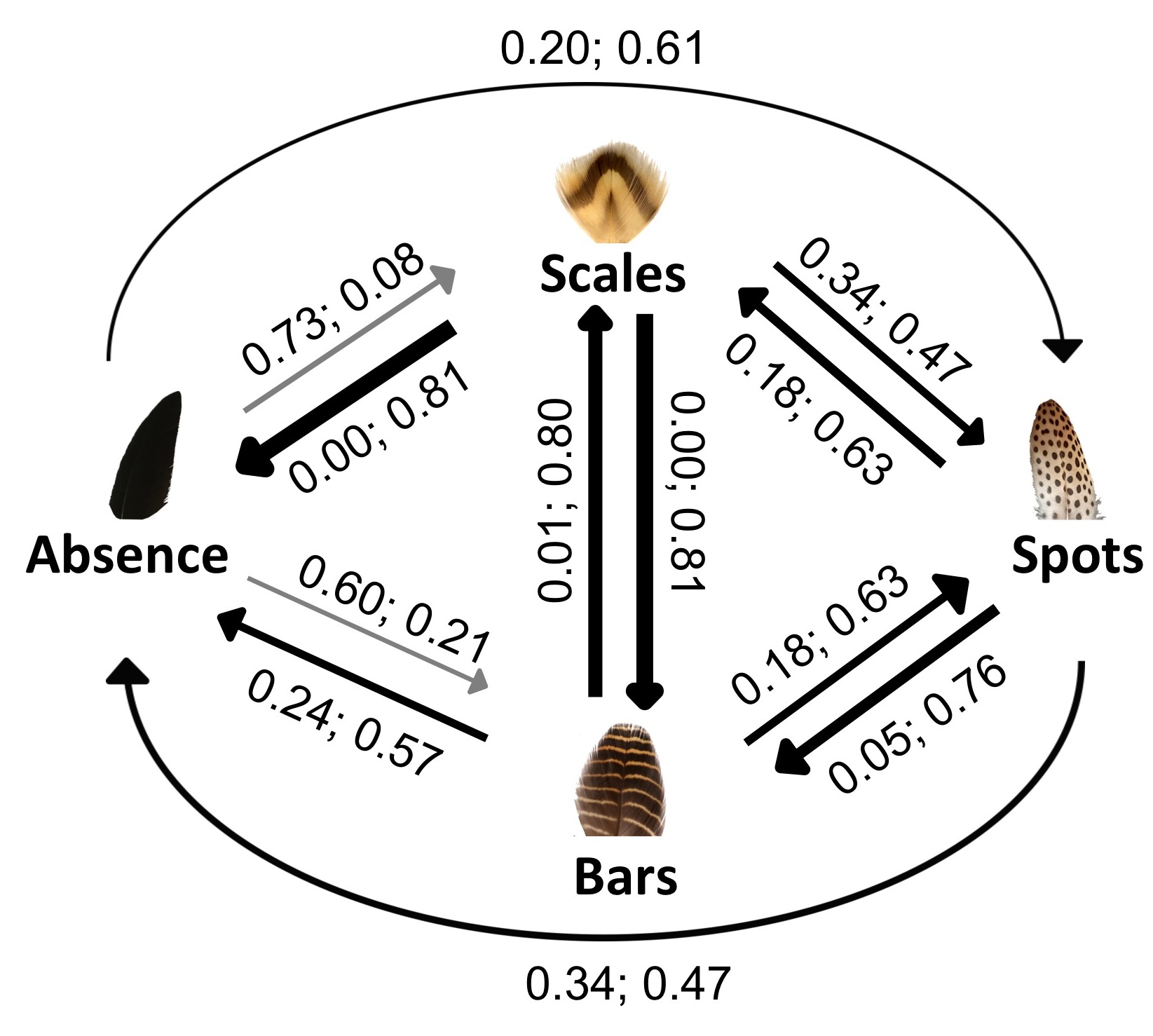
 Flank


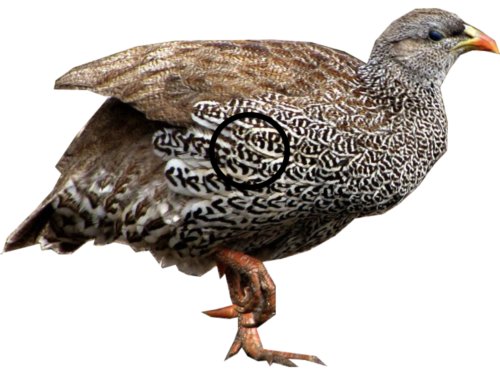

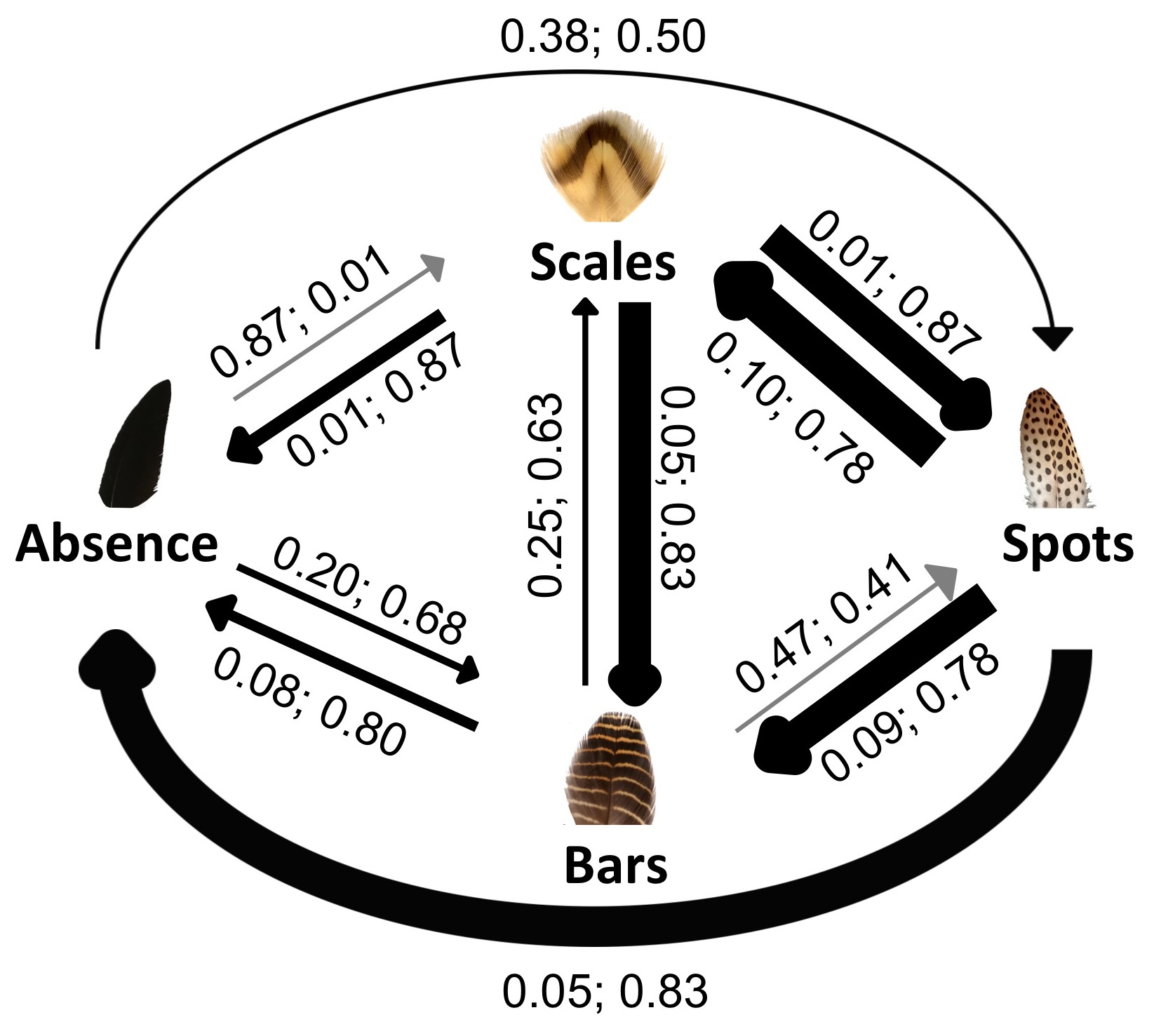


Vent


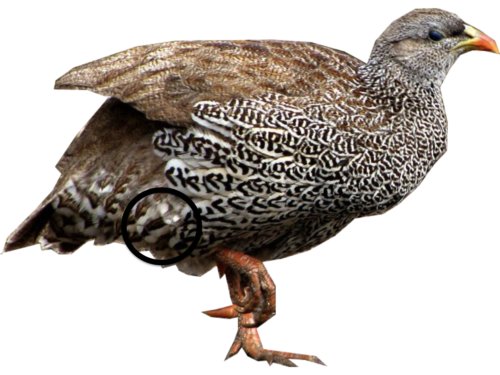

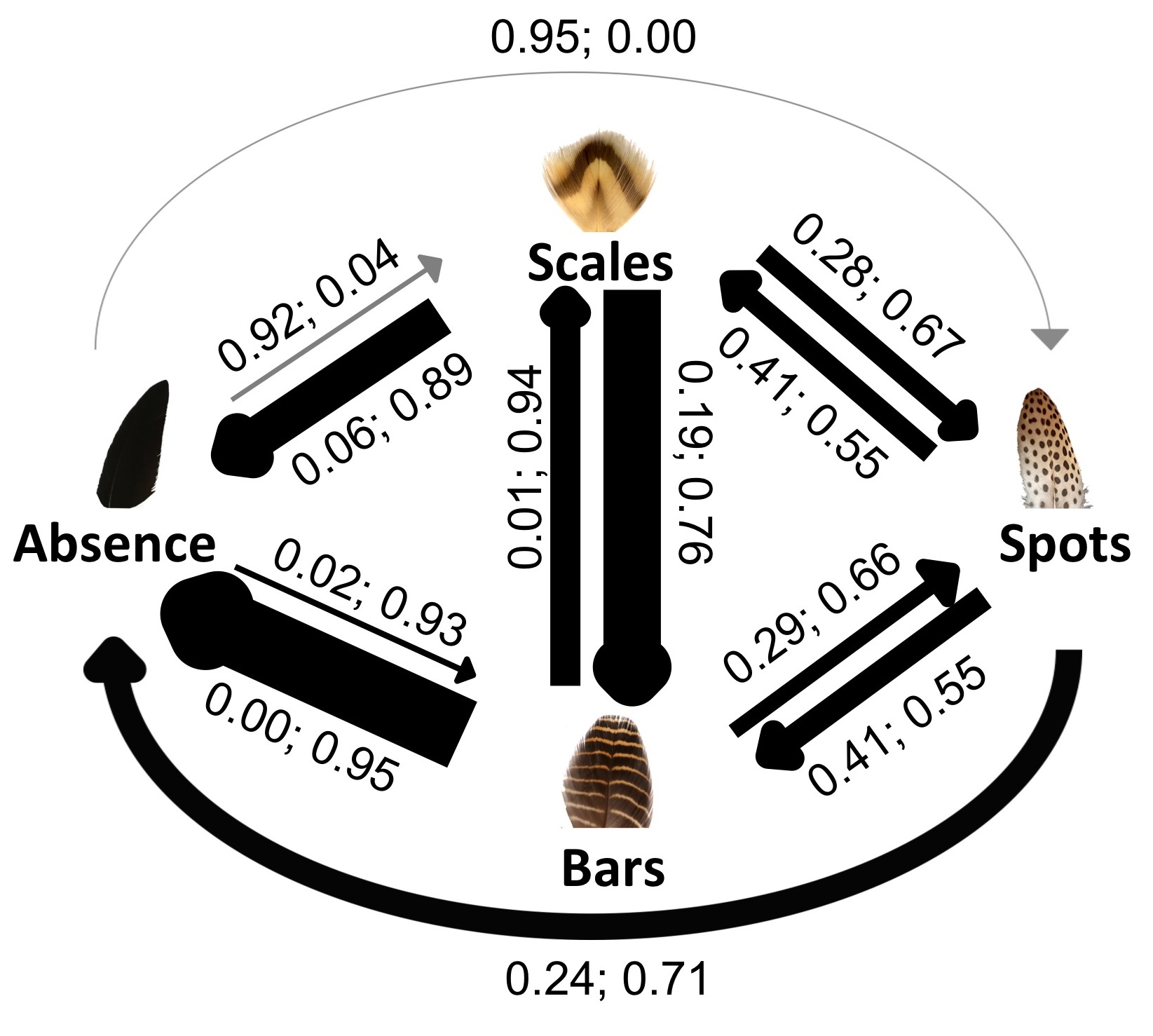


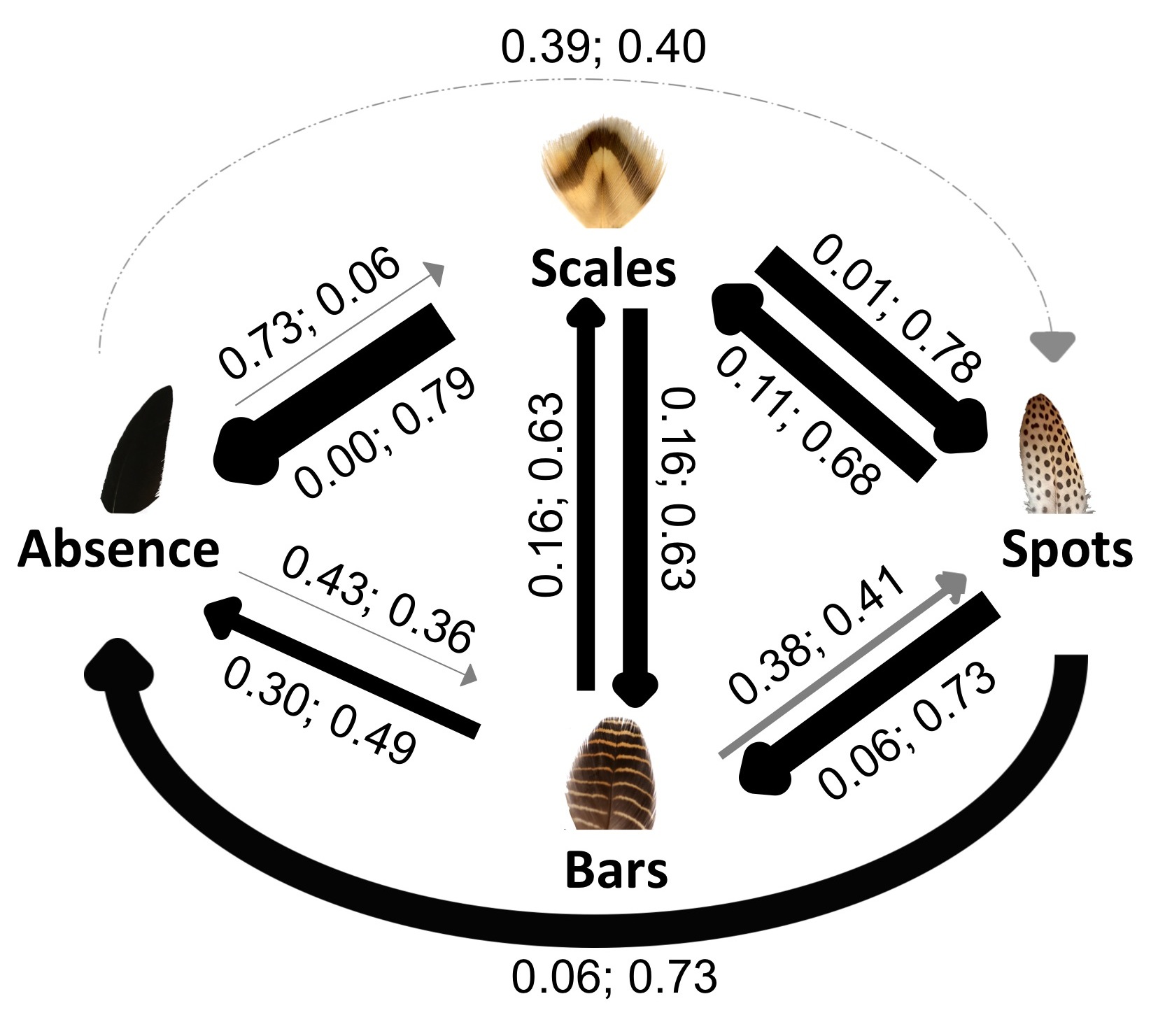


Fig. S2a. Local plumage pattern evolution within individual patches, in Anseriformes and Galliformes using unmodified trees. The width of each evolutionary step is proportional to the average rate per model. Beside each evolutionary step is the marginal probability of each transition not occurring, followed by the marginal probability of it occurring. Where the transition probably does not occur, the transition line is grey. Conversely, where the transition probably does occur, the transition line is black. Equivocal transitions, where the marginal probability is =< 0.05 difference between not occurring and occurring, are indicated by a grey dashed line.

WHOLE BODY

*Scales are derived*


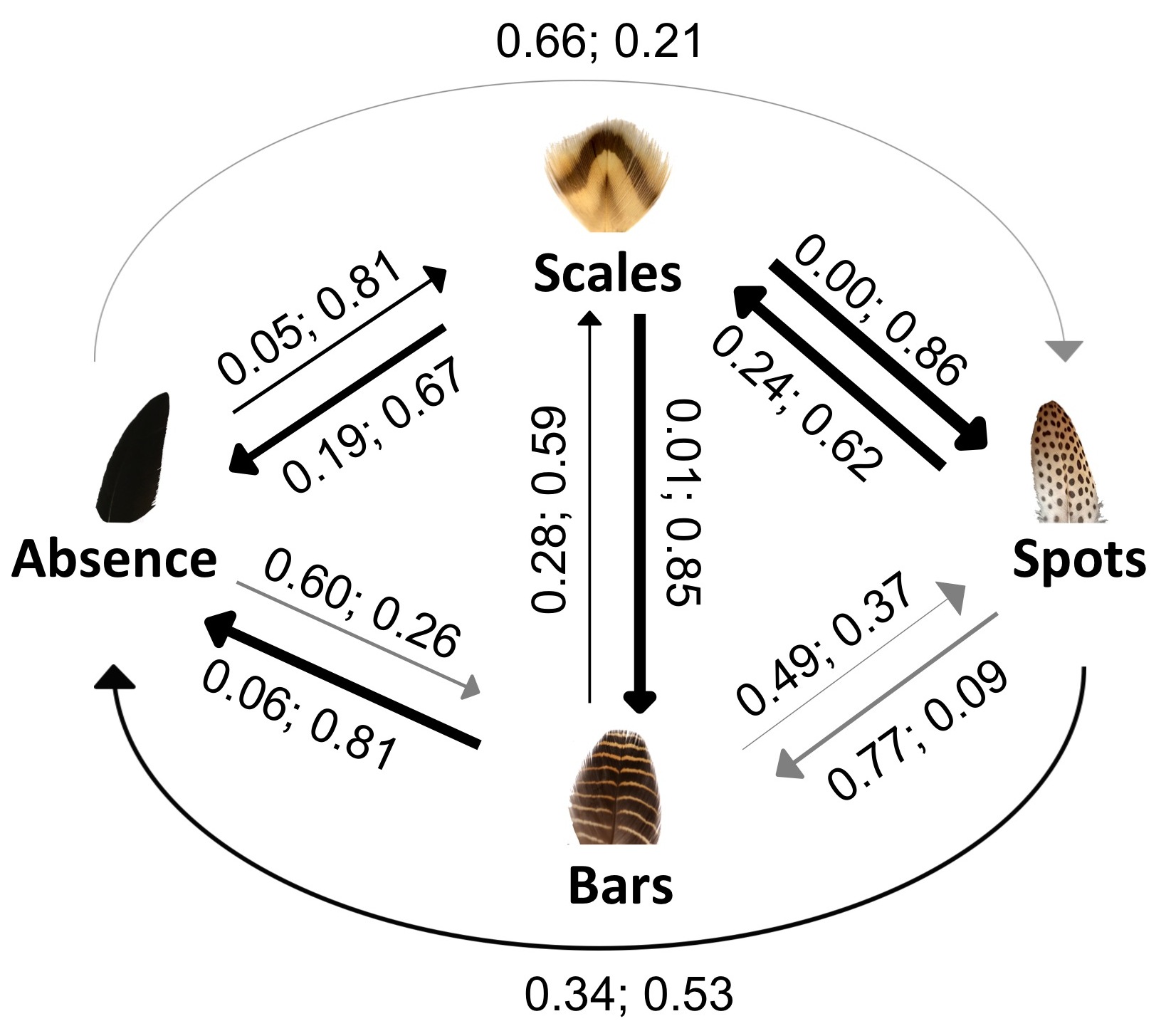

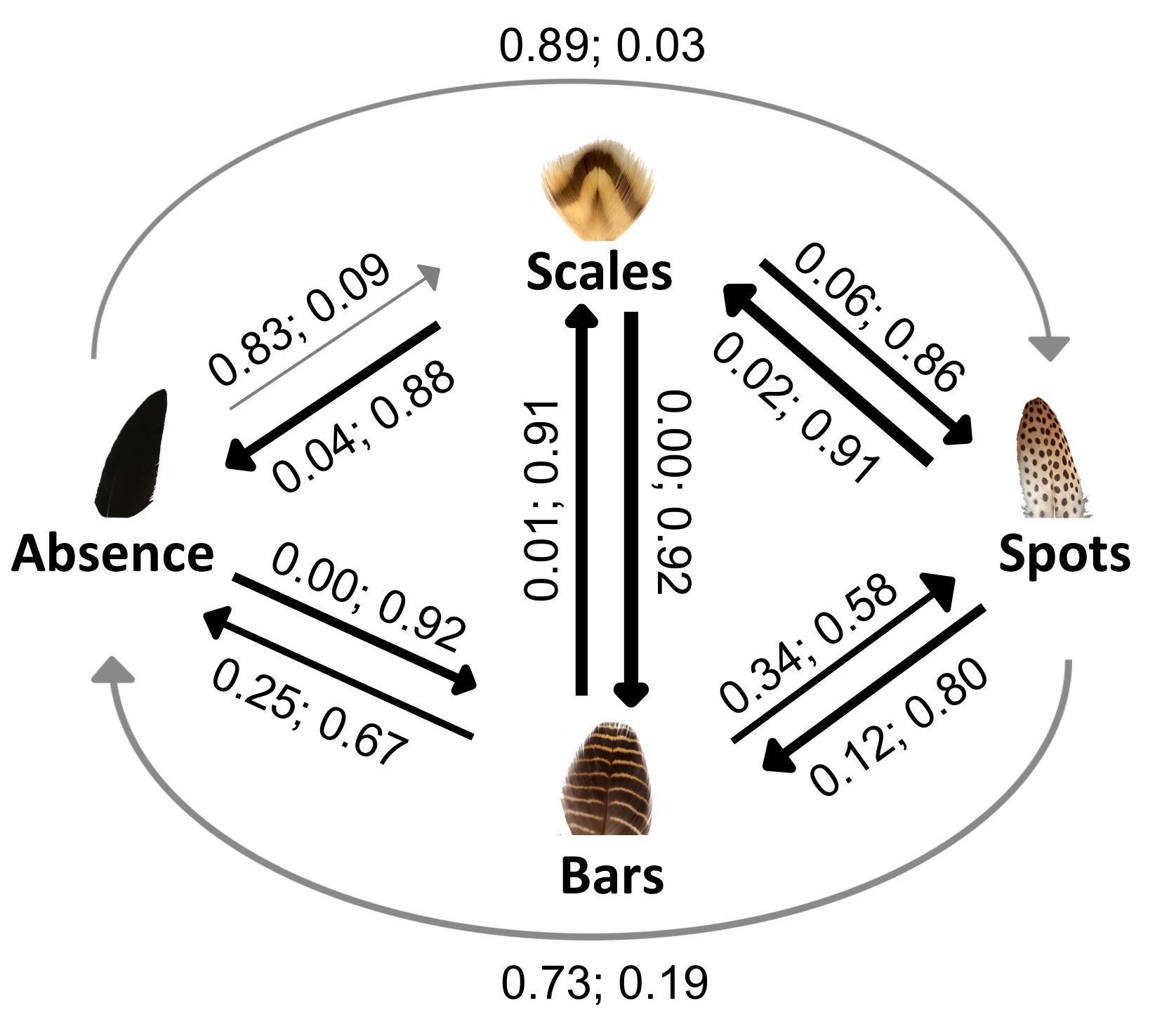
ALL


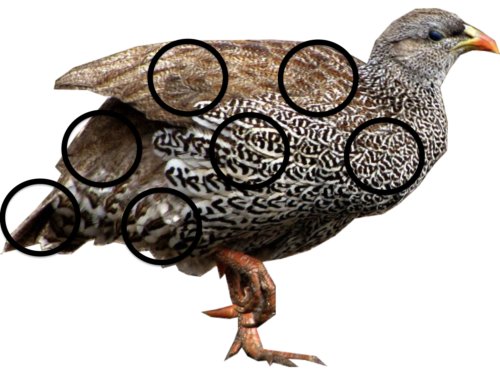


*Spots are derived*


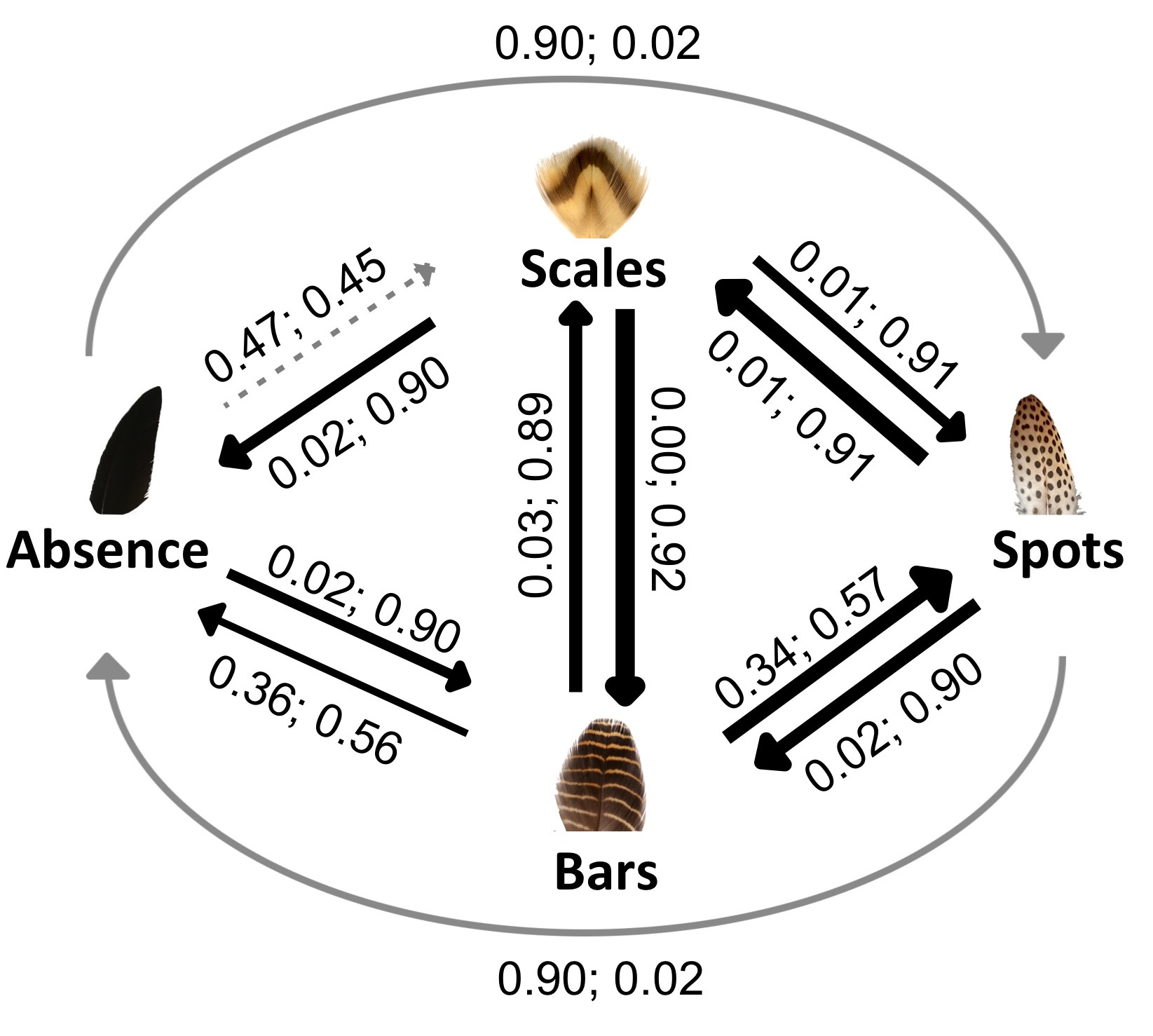


Fig. S2b. Plumage pattern evolution over the whole body, in Anseriformes and Galliformes using unmodified trees. To examine the effects of uncertainty in the order of plumage pattern evolution in Galliformes we modeled the effect of scales or spots being more derived. The width of each evolutionary step is proportional to the average rate in the top model set. Beside each evolutionary step is the marginal probability of each transition not occurring, followed by the marginal probability of it occurring. Where the transition probably does not occur, the transition line is grey. Conversely, where the transition probably does occur, the transition line is black. Equivocal transitions, where the marginal probability is =< 0.05 difference between not occurring and occurring, are indicated by a grey dashed line.

**ANSERIFORMES GALLIFORMES**

UPPERPARTS


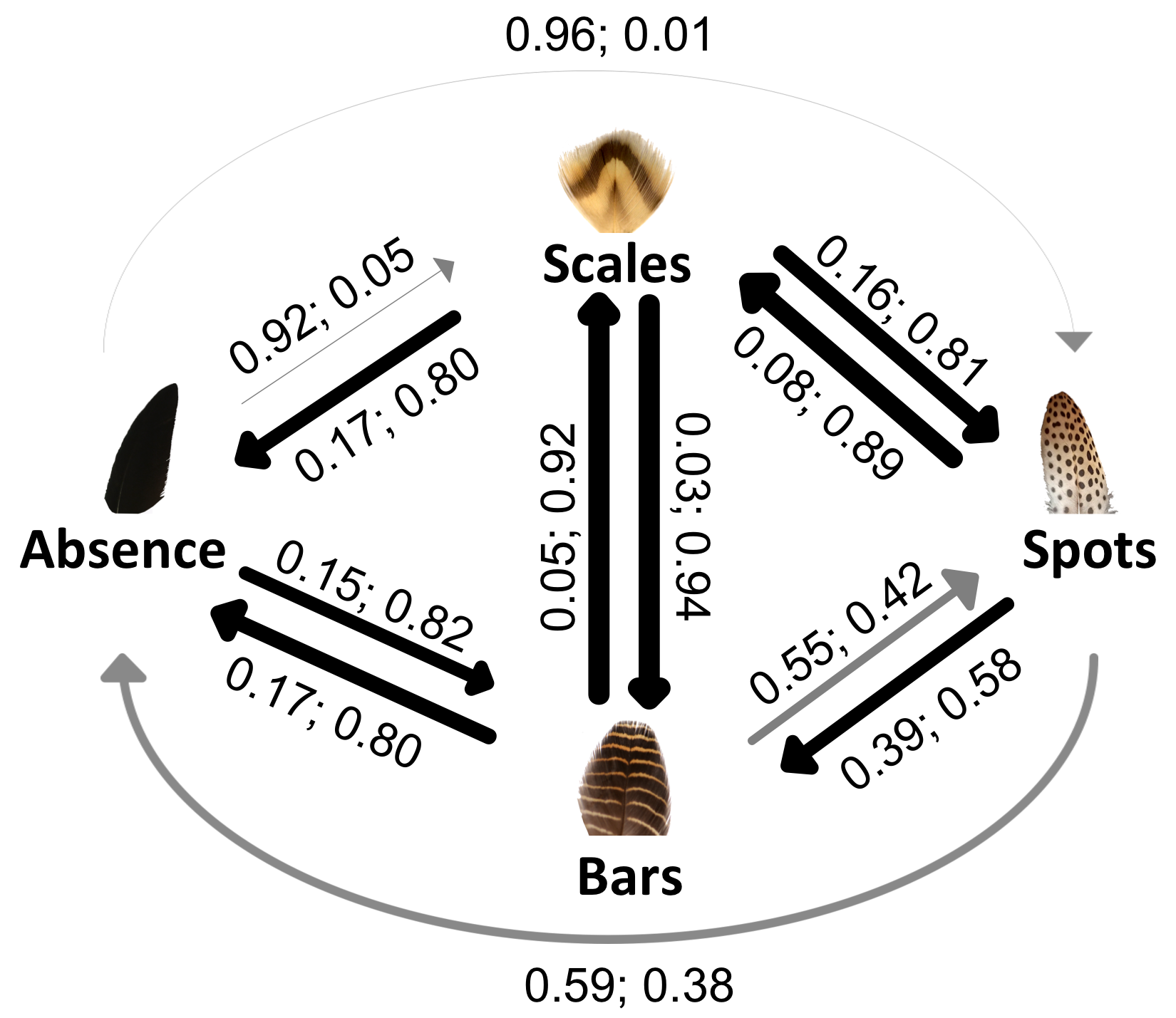
Nape


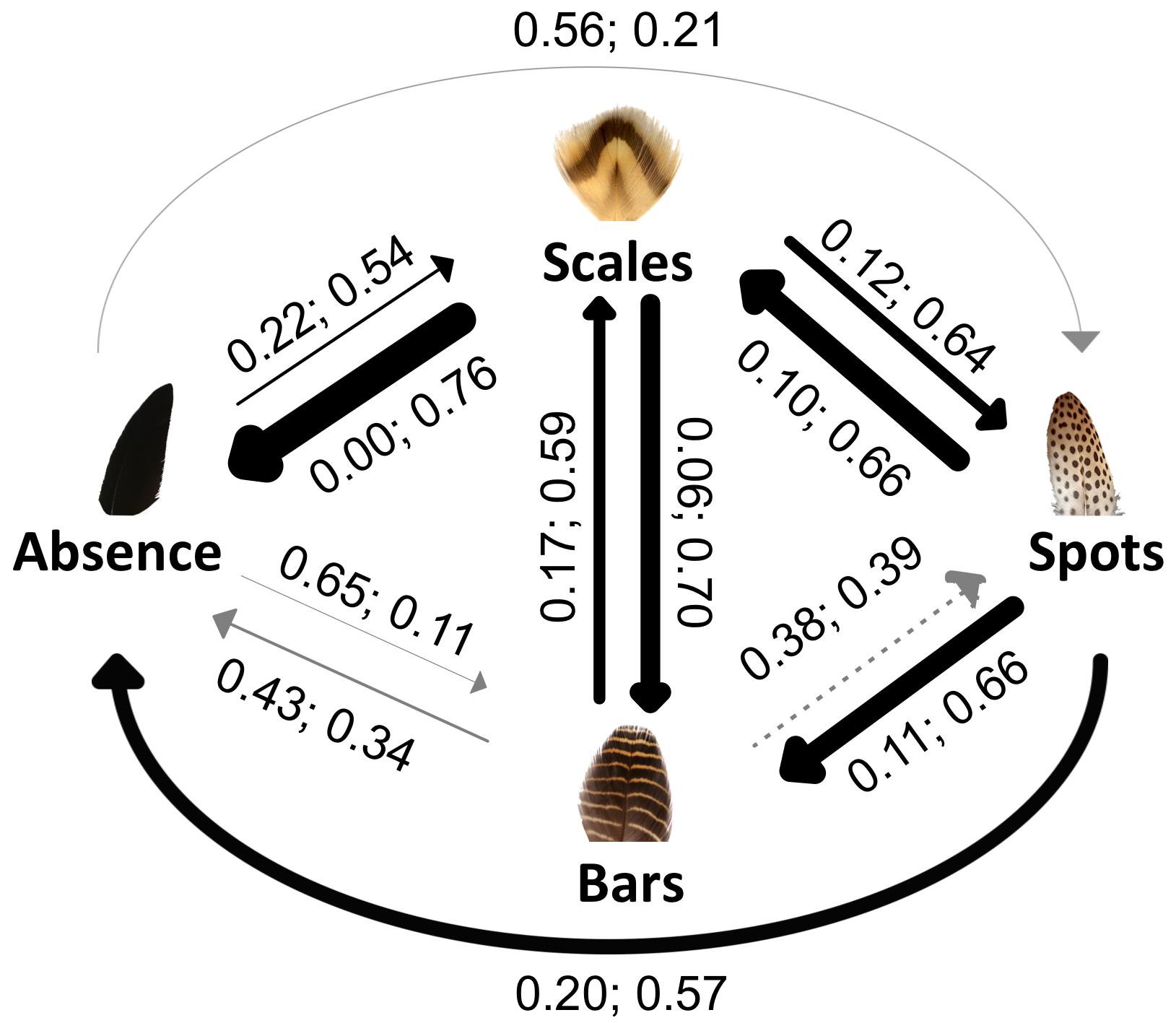

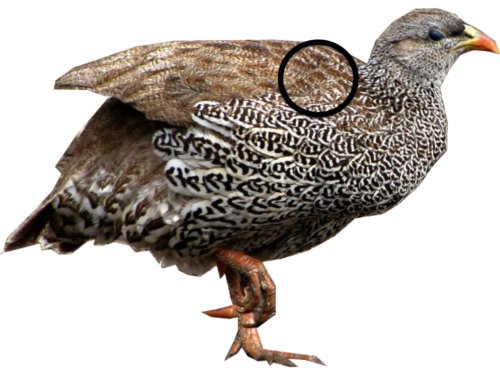


Wing


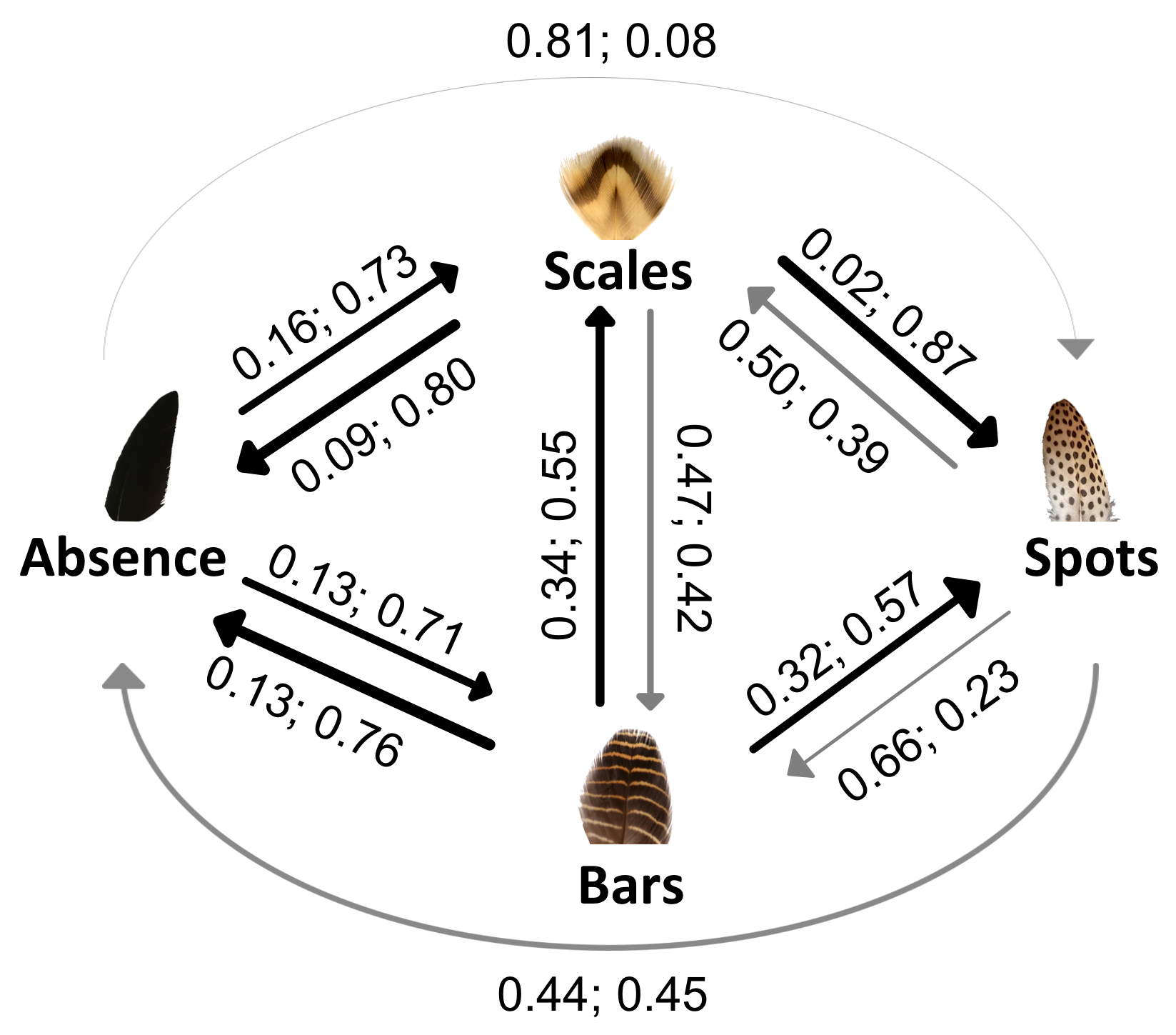

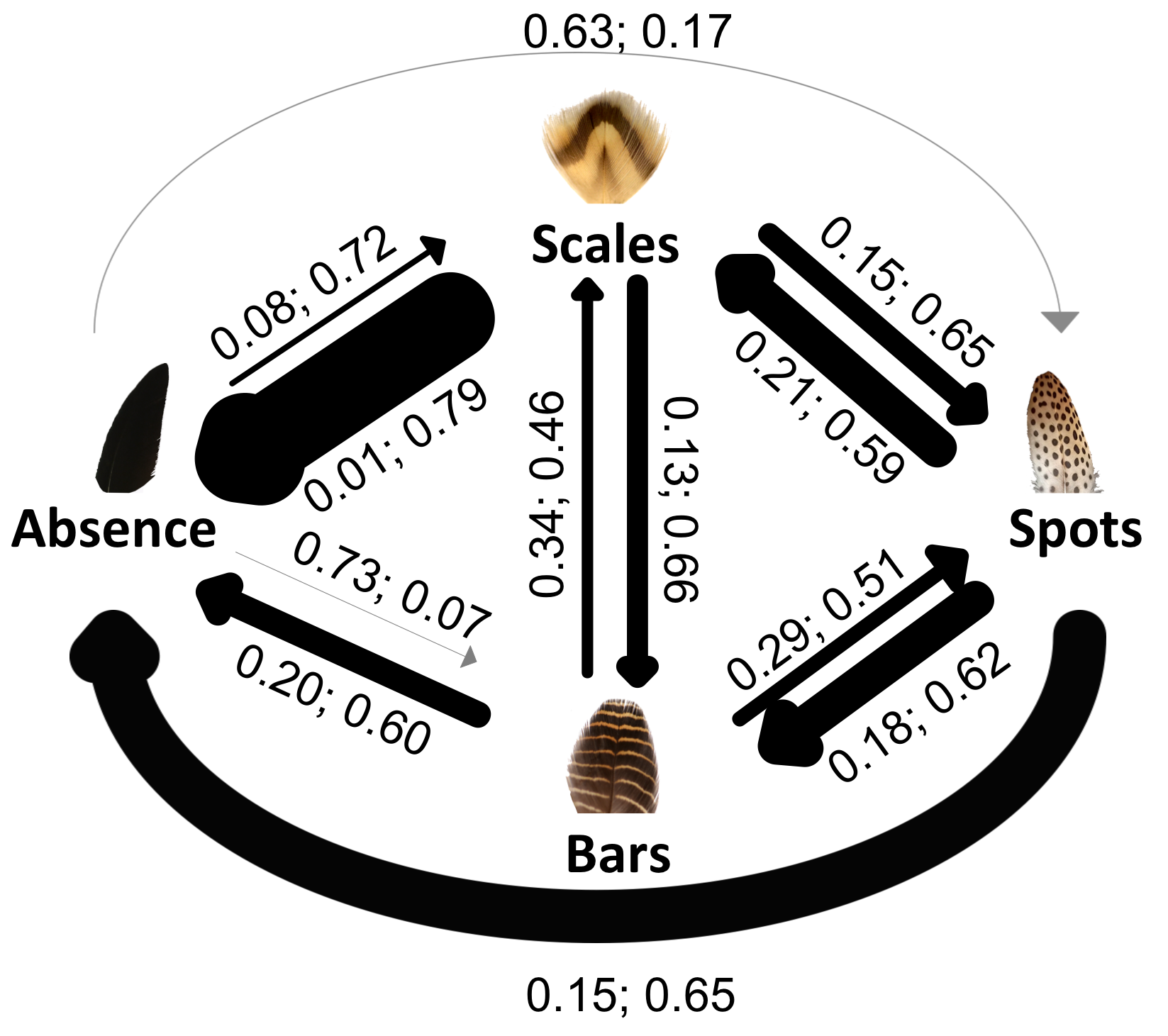

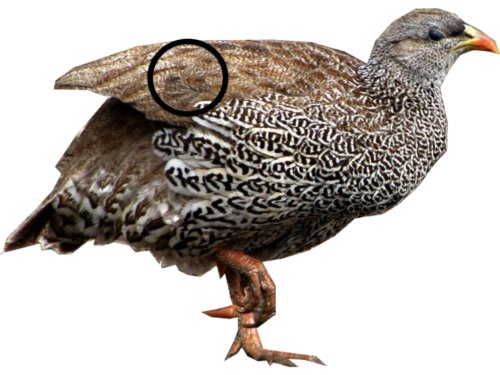


Rump


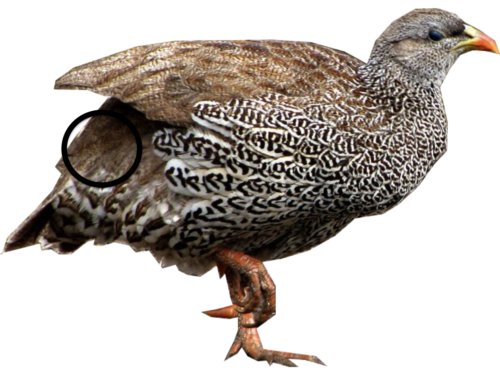


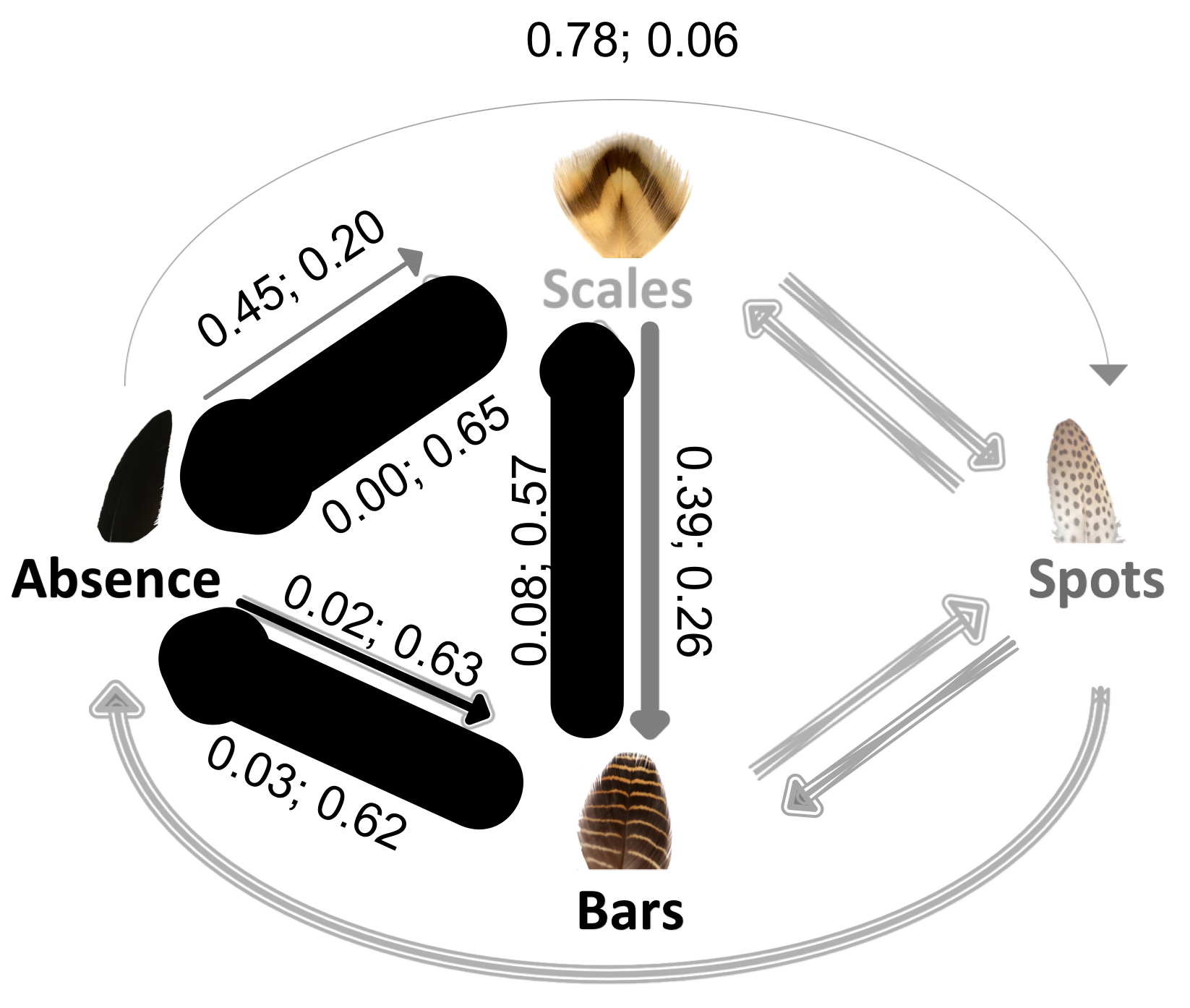


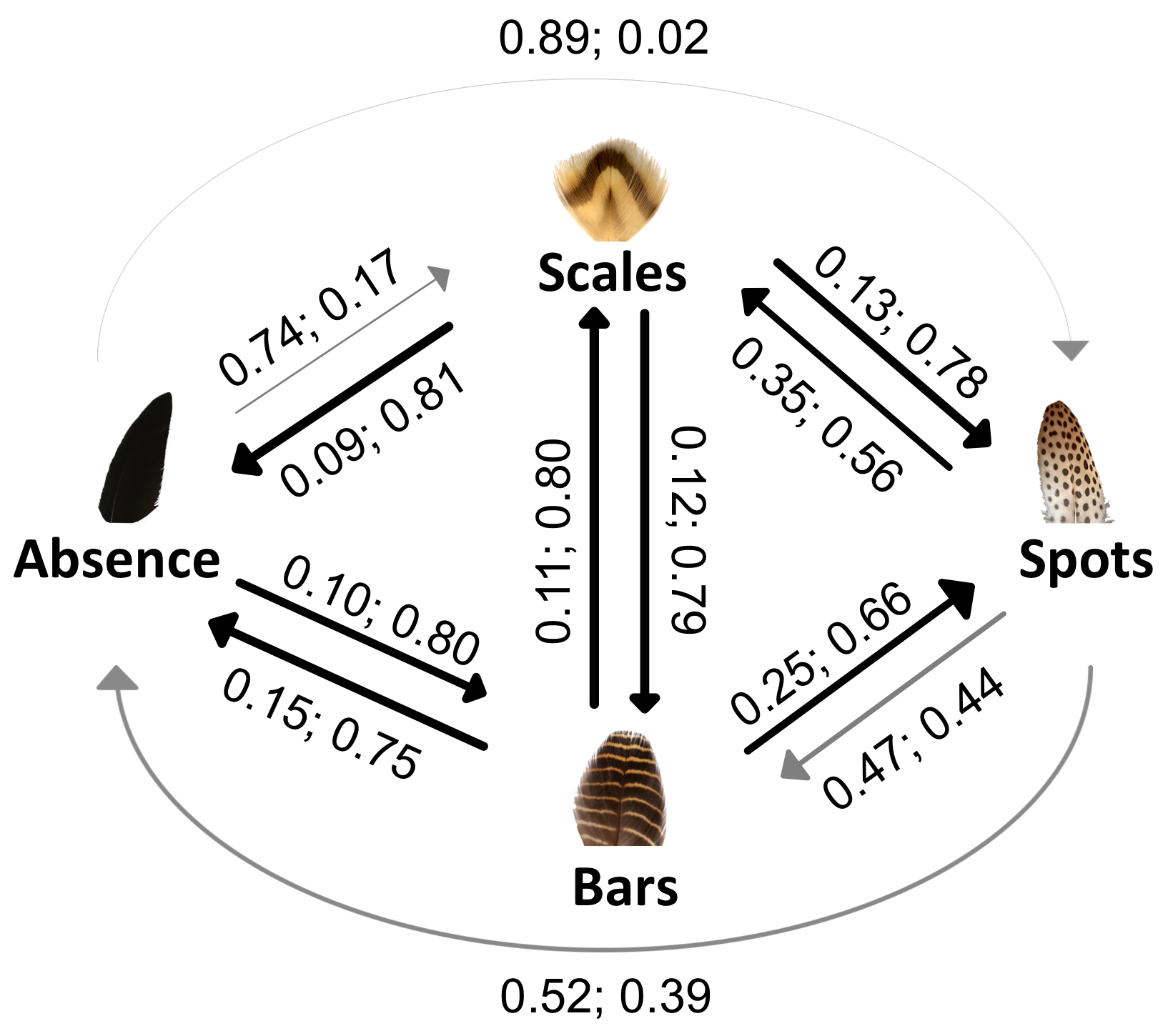


Tail


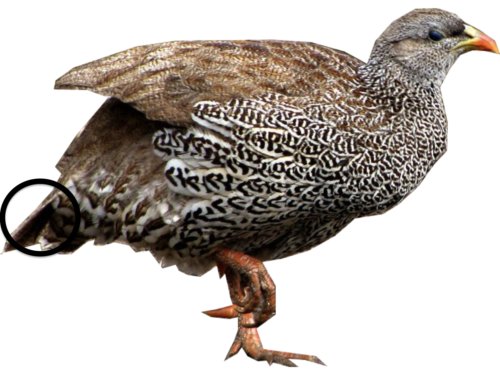


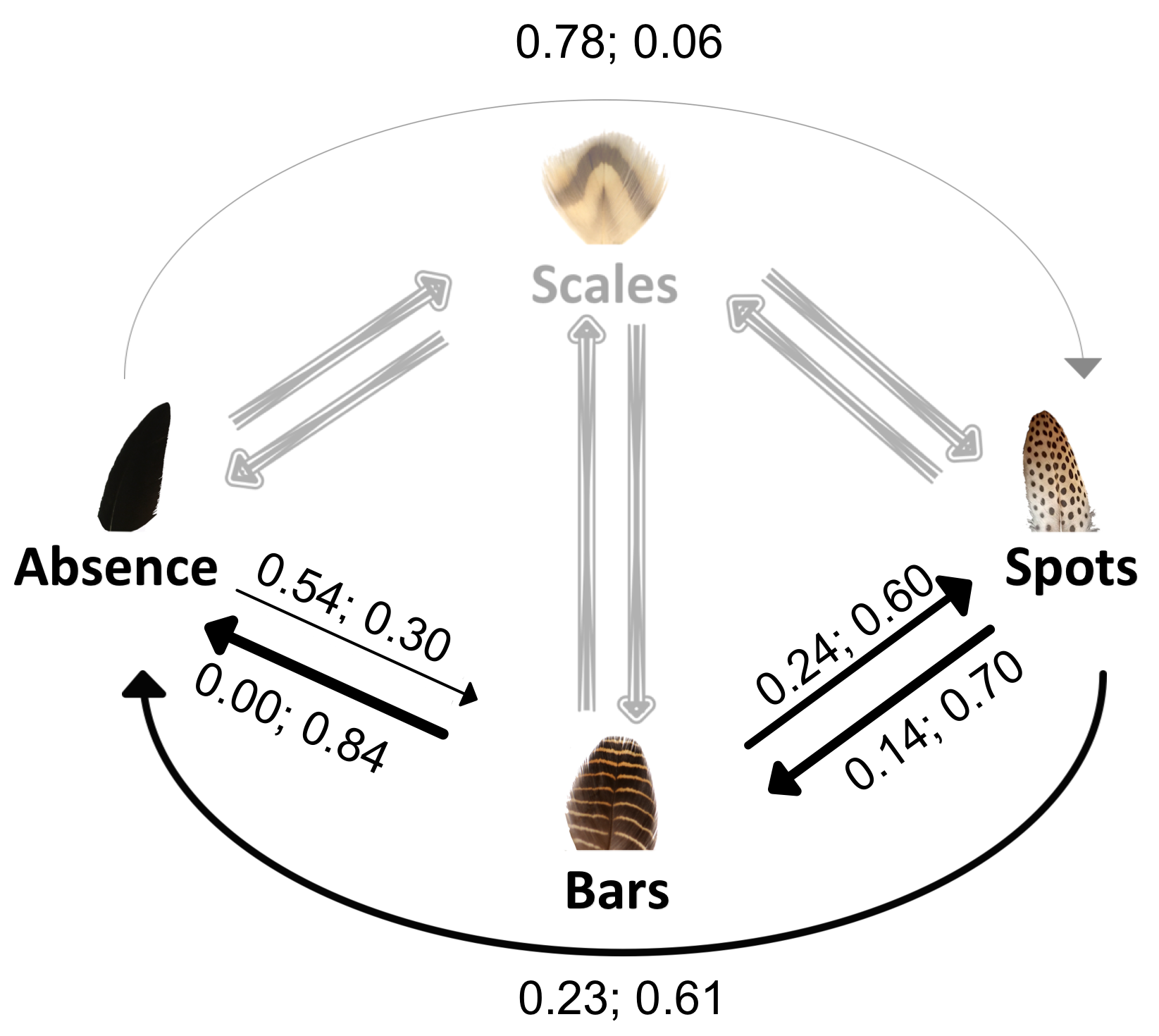


UNDERPARTS

Breast


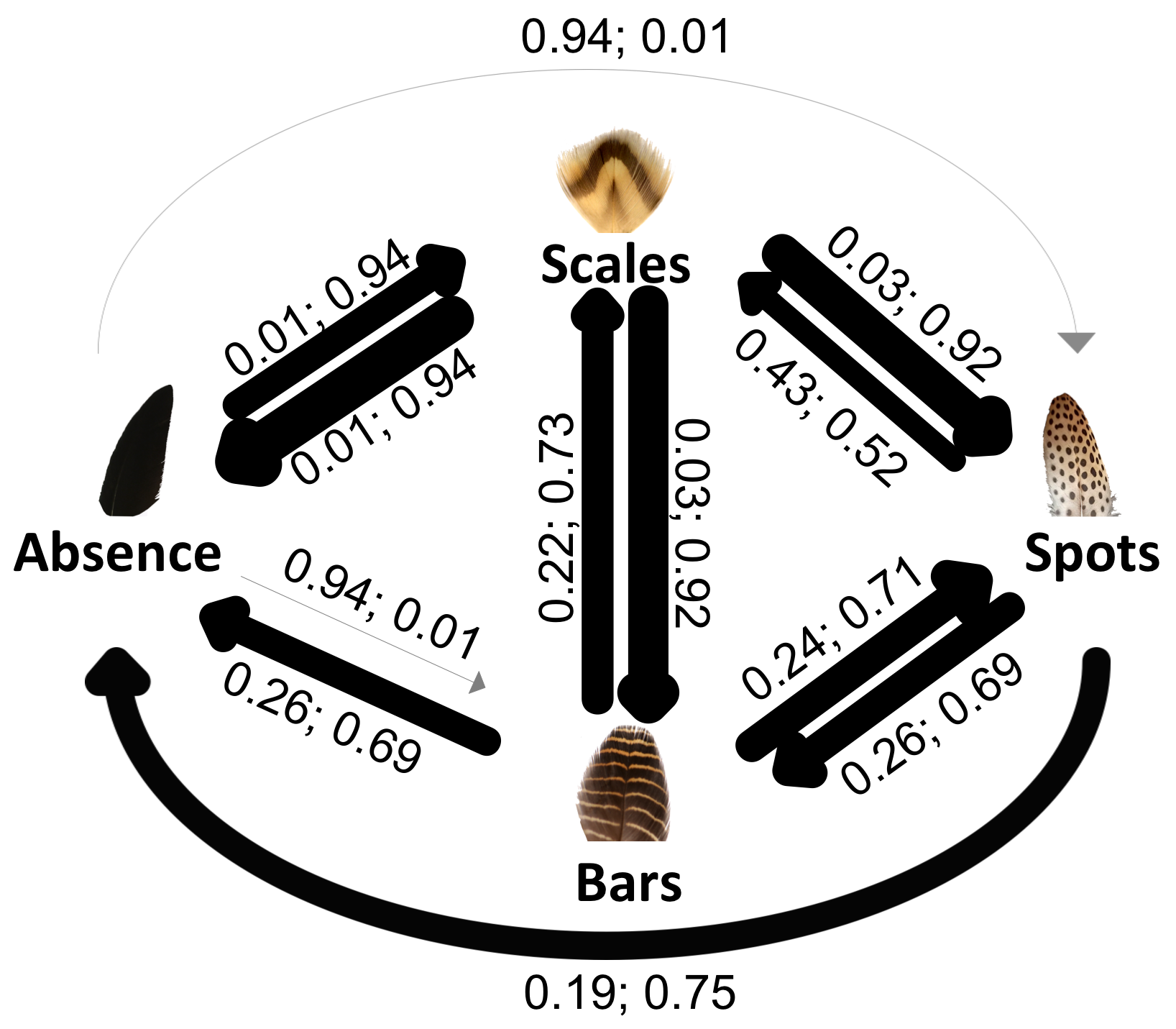


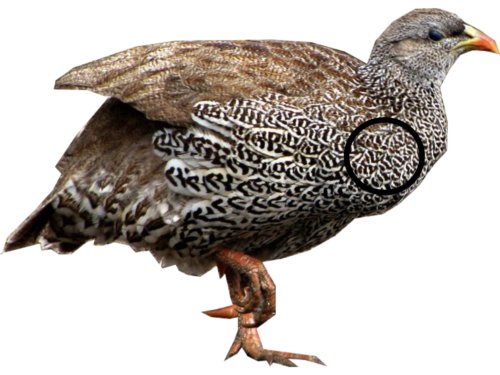


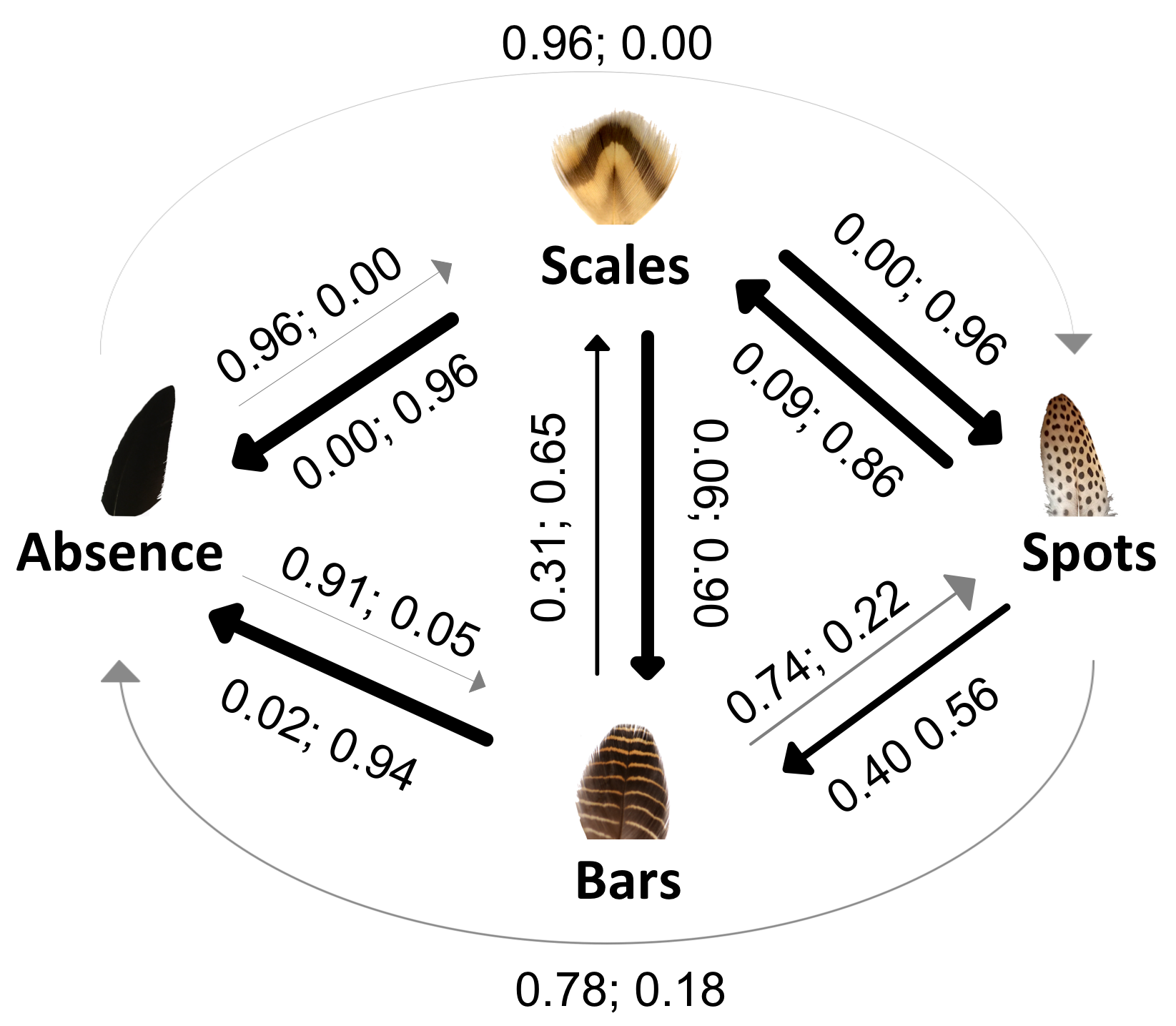


Flank


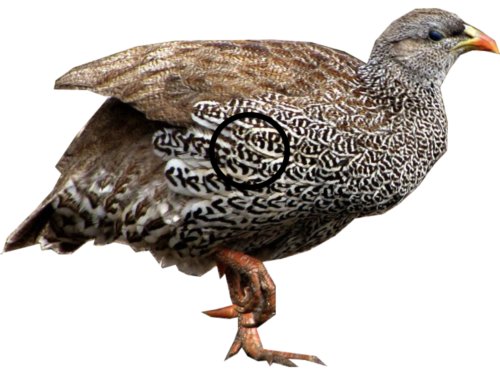


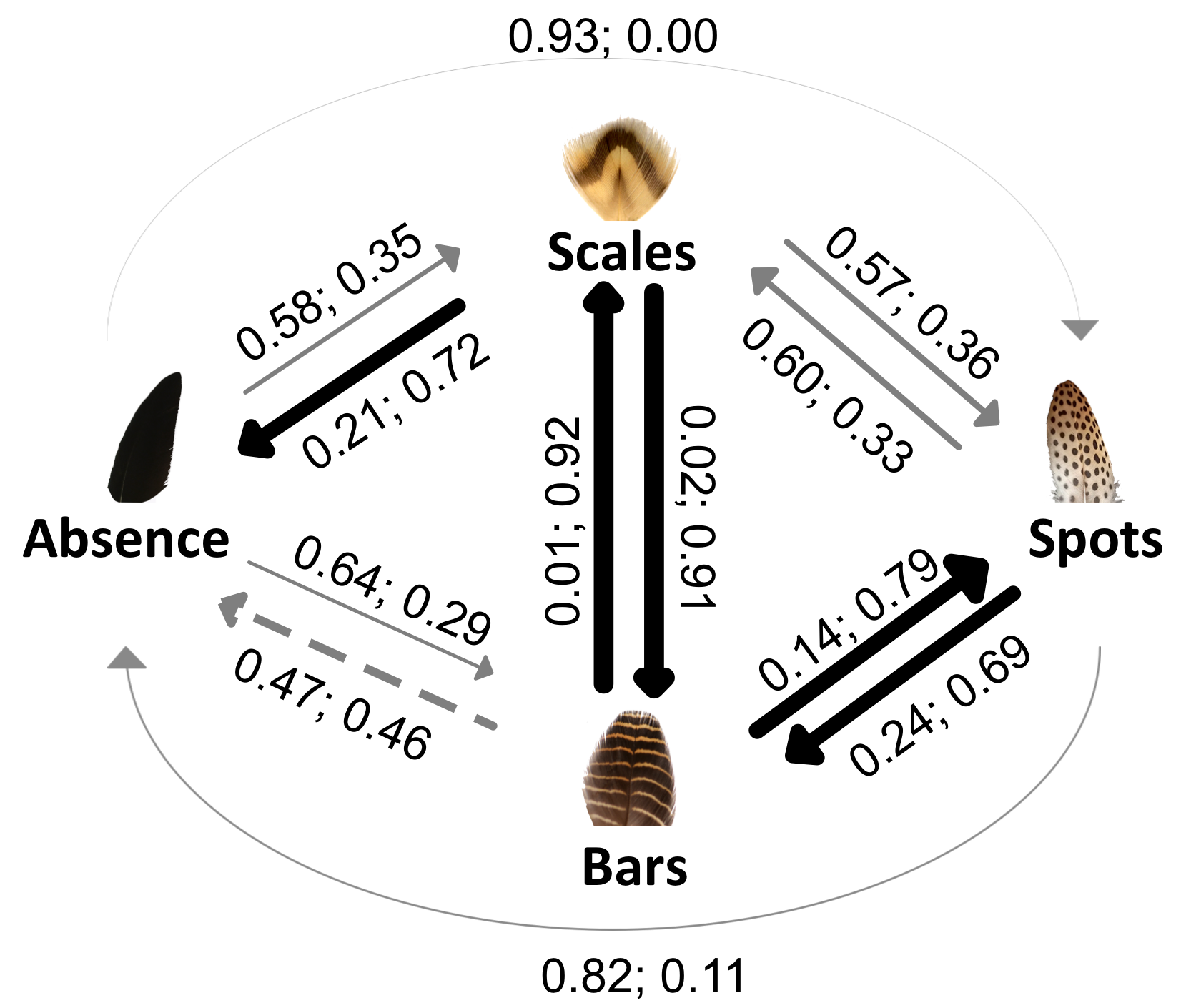

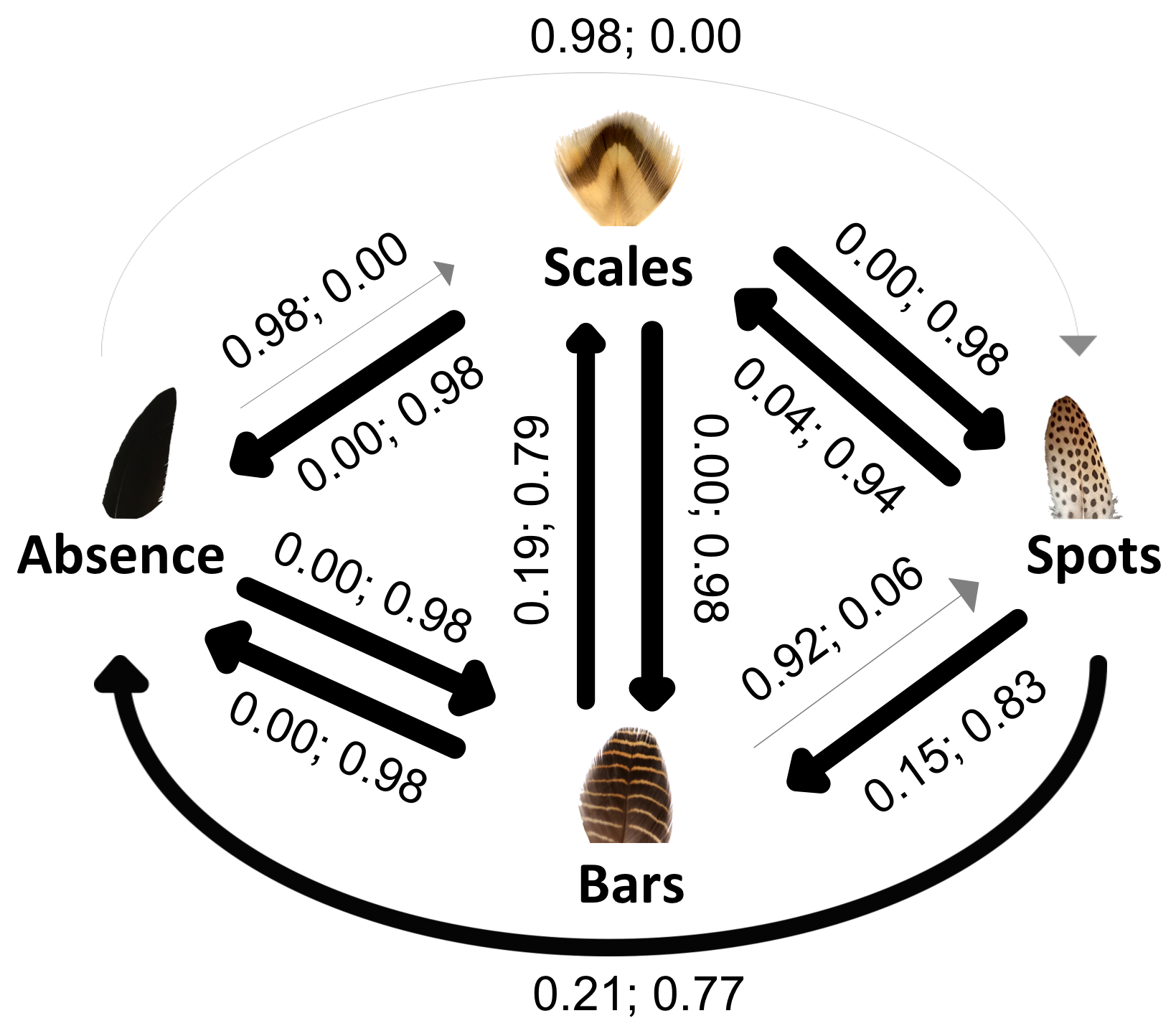


Vent

**
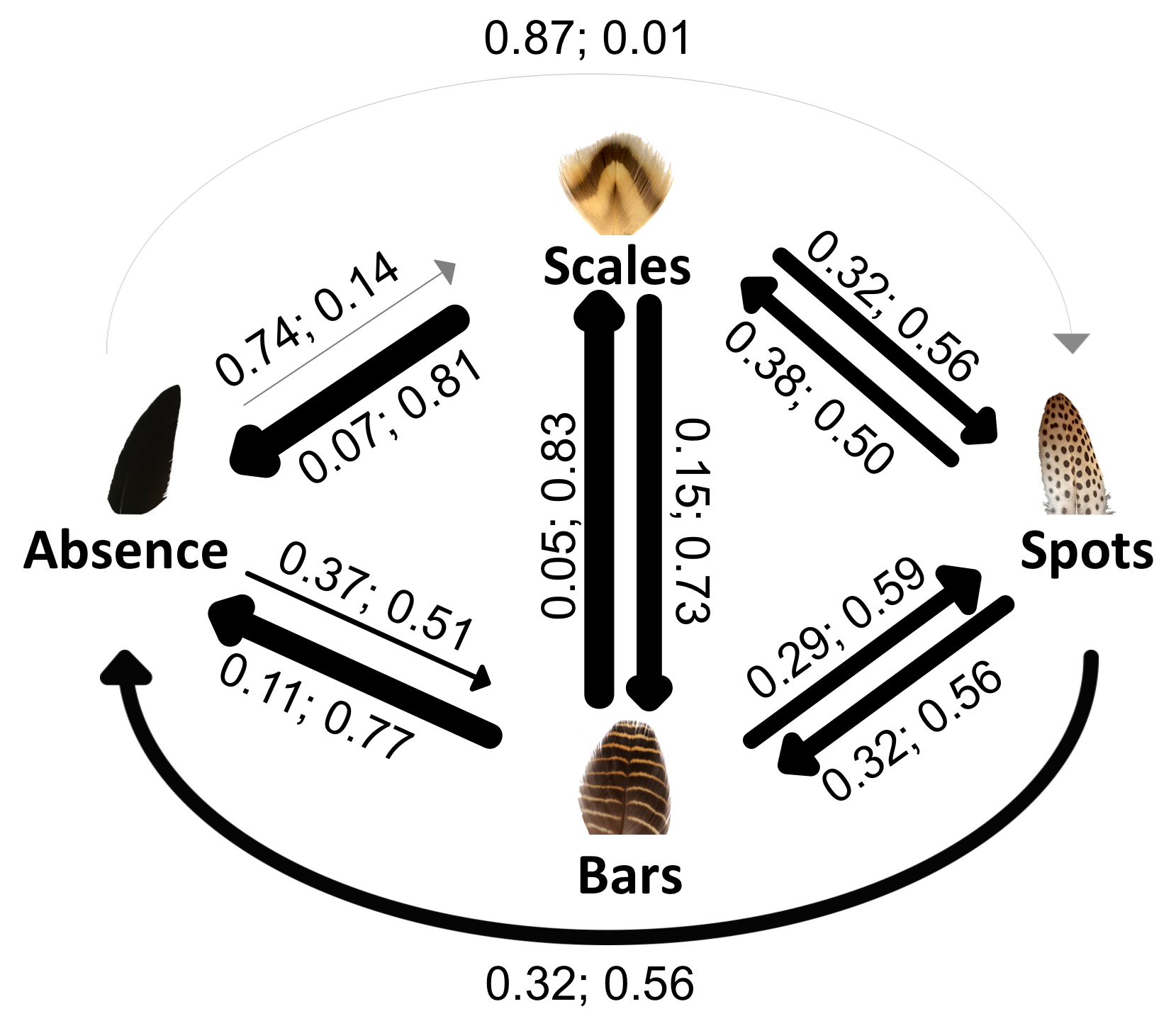
**
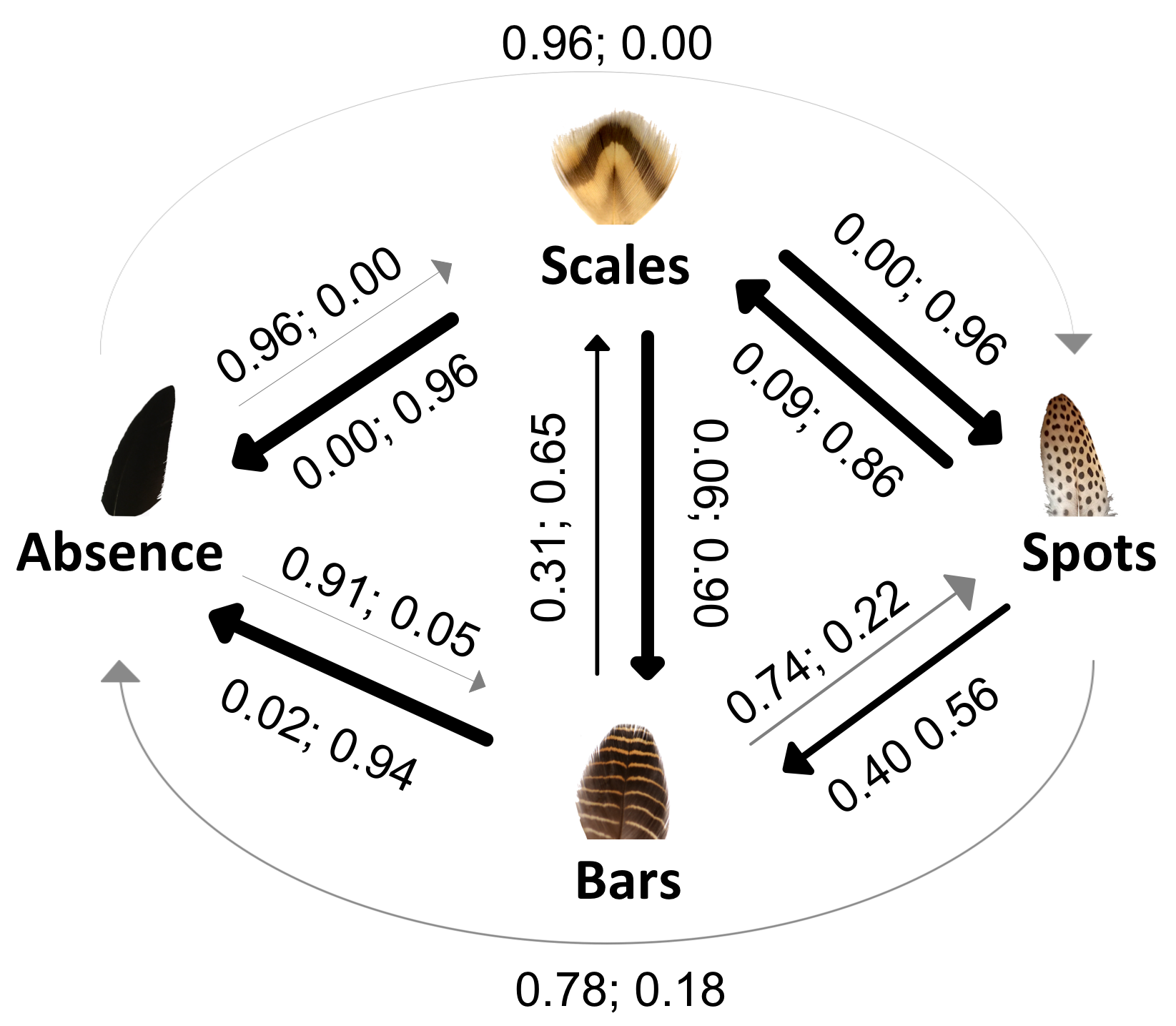

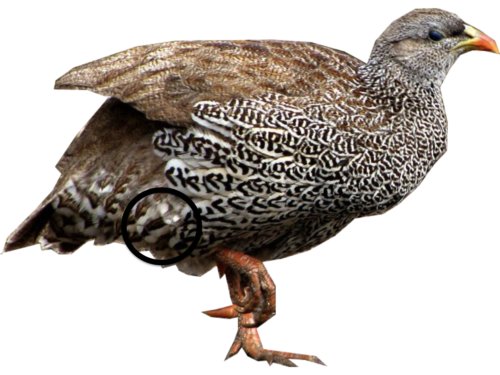


Fig. S3a. Local plumage pattern evolution within individual patches, in Anseriformes and Galliformes using trees that only have branches with a high Bayesian probability. The width of each evolutionary step is proportional to the average rate per model. Beside each evolutionary step is the marginal probability of each transition not occurring, followed by the marginal probability of it occurring. Where the transition probably does not occur, the transition line is grey. Conversely, where the transition probably does occur, the transition line is black. Equivocal transitions, where the marginal probability is =< 0.05 difference between not occurring and occurring, are indicated by a grey dashed line.

WHOLE BODY

*Scales are derived*

*Scales evolved first*


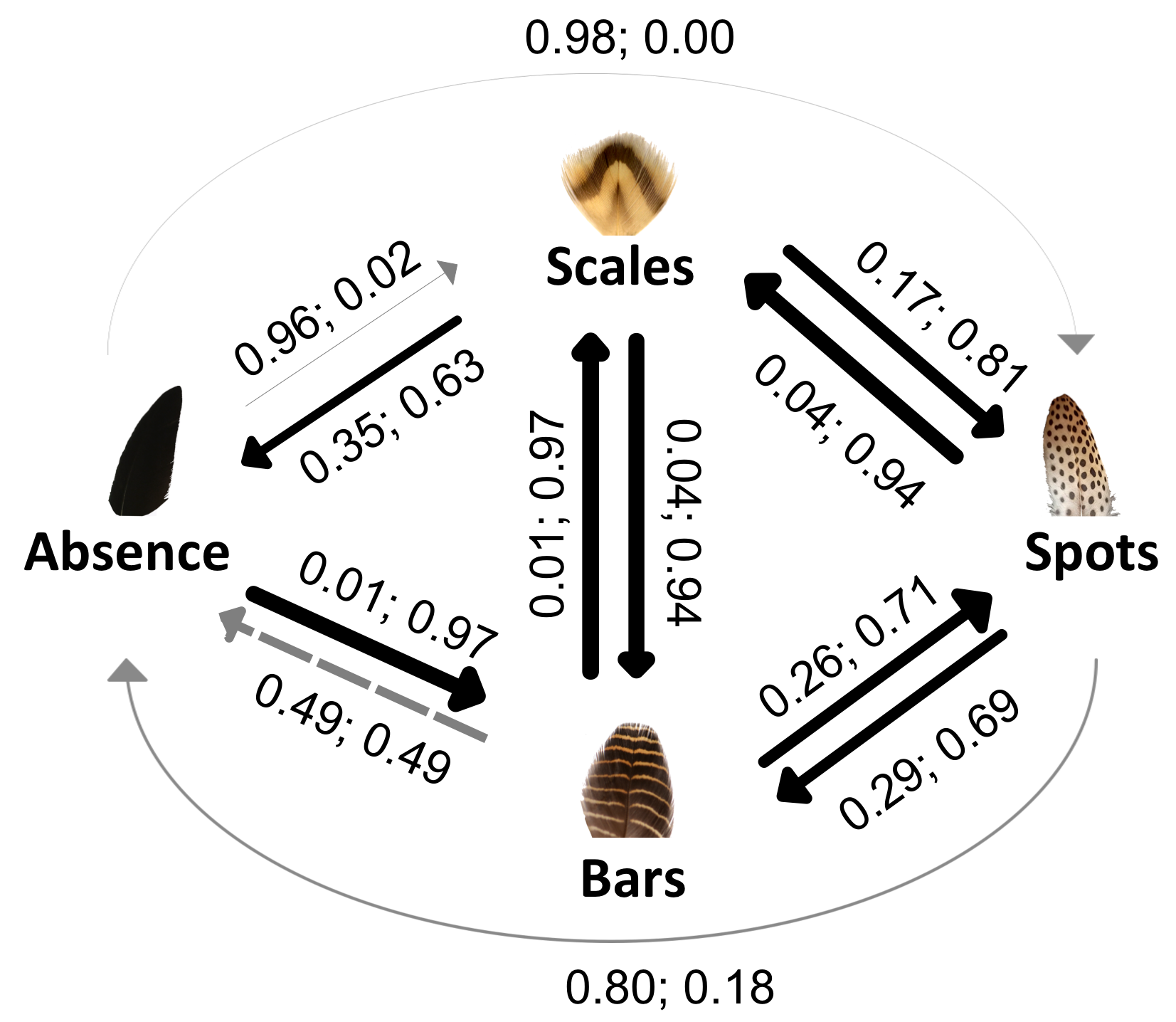
ALL


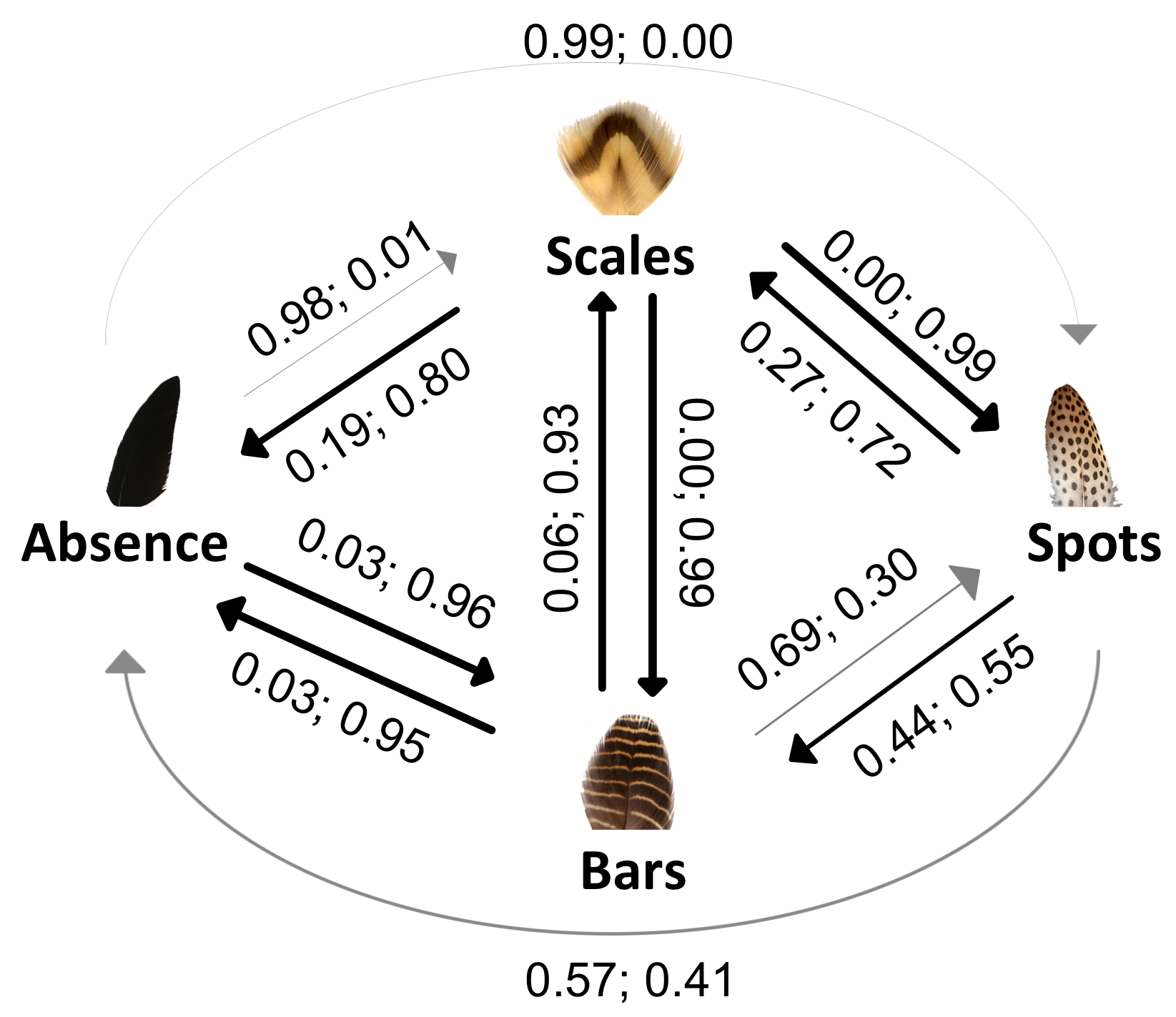

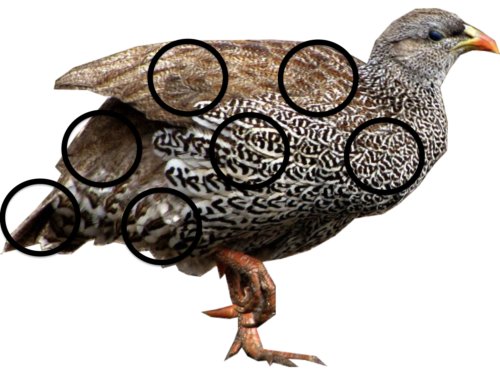


*Bars evolved first*

*Spots are derived*


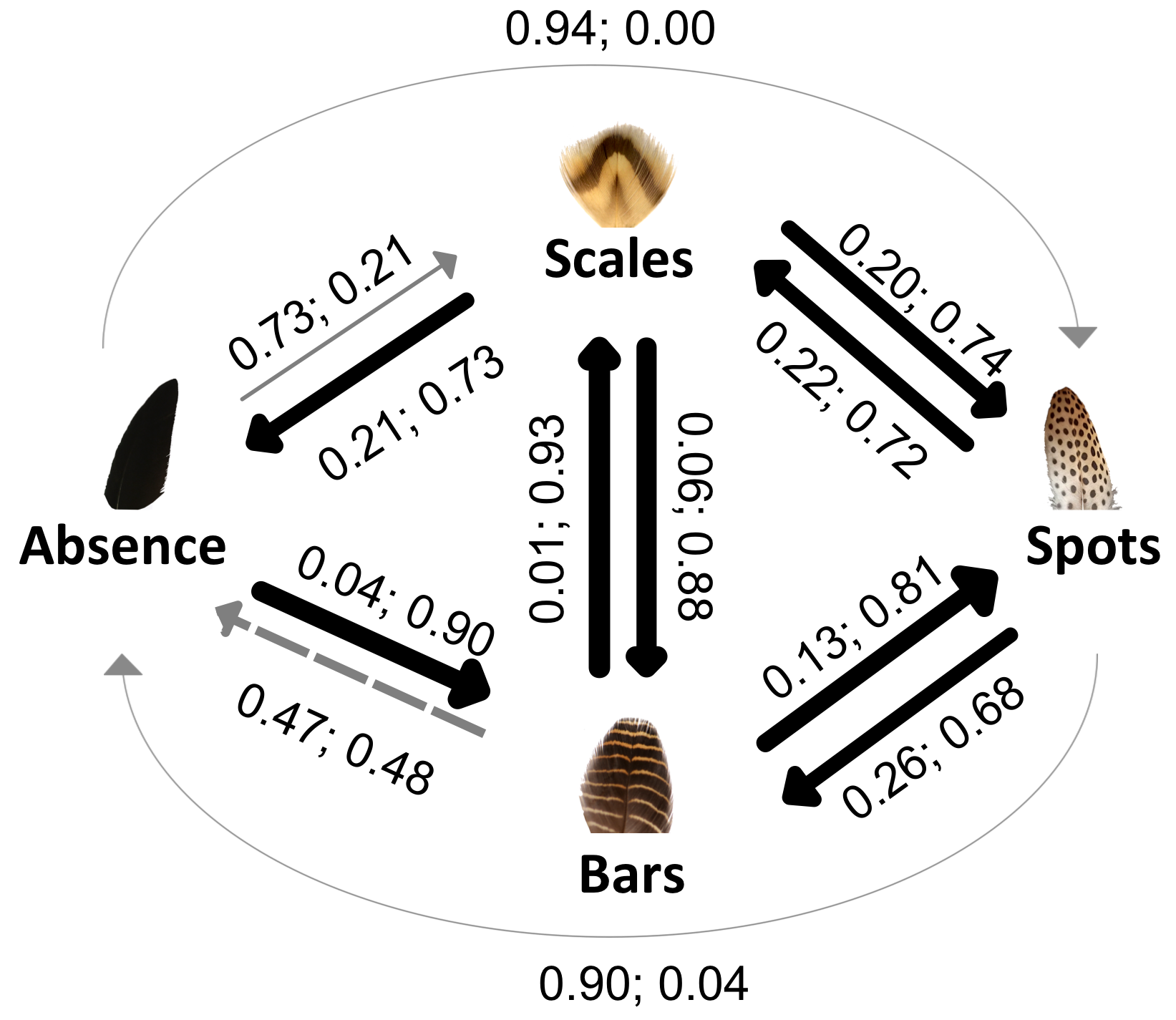

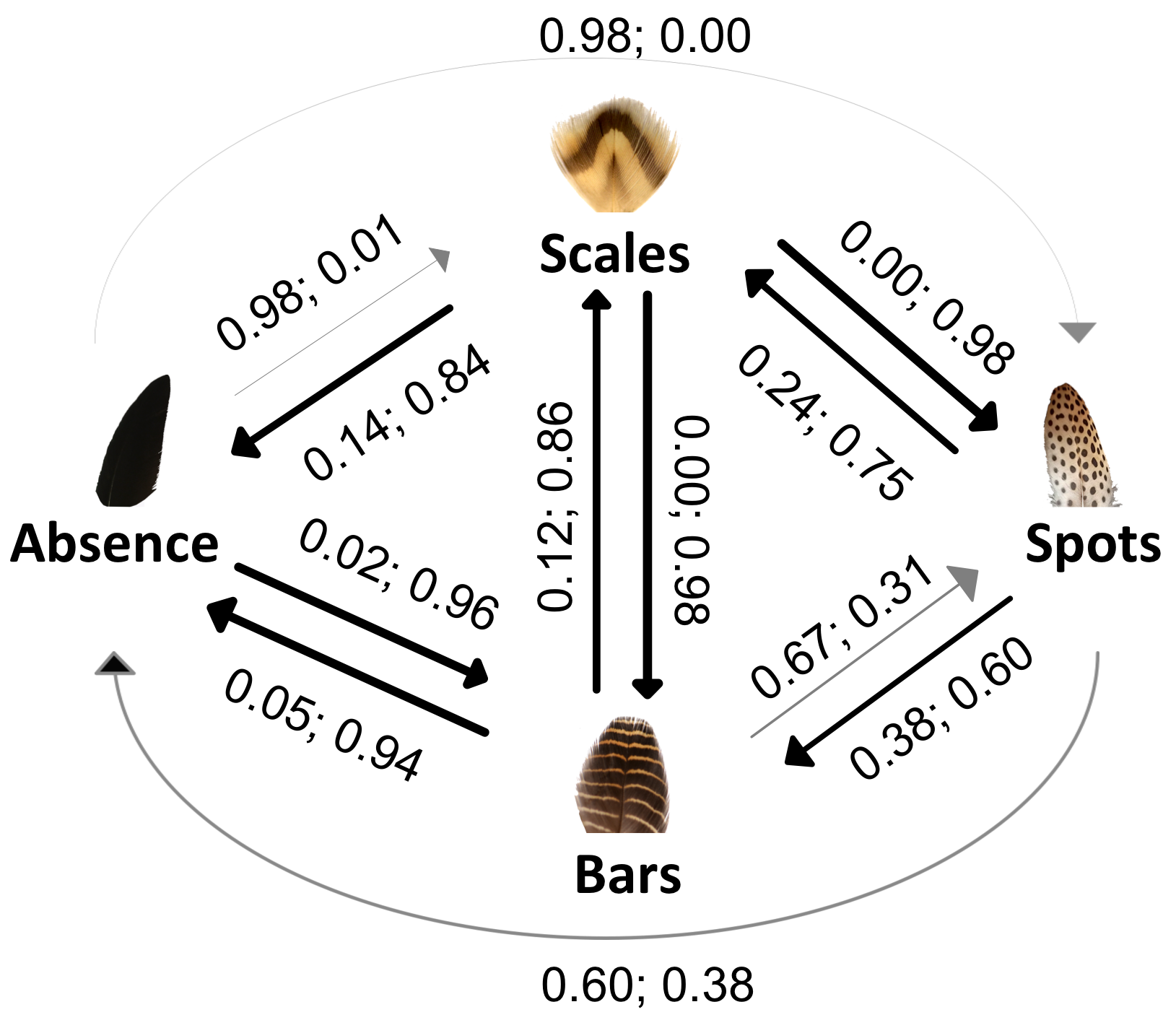


Fig. S3b. Plumage pattern evolution over the whole body, in Anseriformes and Galliformes trees that only have branches with a high Bayesian probability. To examine the effects of uncertainty in the order of plumage pattern evolution in Galliformes we modeled the effect of scales or spots being more derived. The width of each evolutionary step is proportional to the average rate in the top model set. Beside each evolutionary step is the marginal probability of each transition not occurring, followed by the marginal probability of it occurring. Where the transition probably does not occur, the transition line is grey. Conversely, where the transition probably does occur, the transition line is black. Equivocal transitions, where the marginal probability is =< 0.05 difference between not occurring and occurring, are indicated by a grey dashed line.

Table S1. Prior probability of encountering models of n parameters calculated from binomial for Z (where Z = n parameters that are set to 0 i.e. do not occur) and Bell numbers for models with 12 possible transition rates.

| No. Zeros (z) | Binomial for z | 12 - z | Bell number for 12 - z | Binomial for z x bell number for 12 - z | Prior |
| --- | --- | --- | --- | --- | --- |
| 0 | 1 | 12 | 4213597 | 4213597 | 0.1524211599 |
| 1 | 12 | 11 | 678570 | 8142840 | 0.0245463499 |
| 2 | 66 | 10 | 115975 | 7654350 | 0.0041952384 |
| 3 | 220 | 9 | 21147 | 4652340 | 0.0007649641 |
| 4 | 495 | 8 | 4140 | 2049300 | 0.0001497589 |
| 5 | 792 | 7 | 877 | 694584 | 0.0000317243 |
| 6 | 924 | 6 | 203 | 187572 | 0.0000073432 |
| 7 | 792 | 5 | 52 | 41184 | 0.0000018810 |
| 8 | 495 | 4 | 15 | 7425 | 0.0000005426 |
| 9 | 220 | 3 | 5 | 1100 | 0.0000001809 |
| 10 | 66 | 2 | 2 | 132 | 0.0000000723 |
| 11 | 12 | 1 | 1 | 12 | 0.0000000362 |

Table S2. Prior probability of encountering models of n parameters calculated from binomial for Z (where Z = n parameters that are set to 0 i.e. do not occur) and Bell numbers for models with three pattern states encompassing 6 possible transitions.

| No of zeroes | Binomial for z | 6-z | Bell number for 6-z | Binomial for z x bell number for 6-z | Prior |
| --- | --- | --- | --- | --- | --- |
| 0 | 1 | 6 | 203 | 203 | 0.23173516 |
| 1 | 6 | 5 | 52 | 312 | 0.04577465 |
| 2 | 15 | 4 | 15 | 225 | 0.01381216 |
| 3 | 20 | 3 | 5 | 100 | 0.00514933 |
| 4 | 15 | 2 | 2 | 30 | 0.00221239 |
| 5 | 6 | 1 | 1 | 6 | 0.00113507 |

**References**

1. Gonzalez J, Düttmann H, Wink M. Phylogenetic relationships based on two mitochondrial genes and hybridization patterns in Anatidae. Journal of Zoology. 2009;279:310–8.

2. Kimball RT, Mary CMS, Braun EL. A Macroevolutionary Perspective on Multiple Sexual Traits in the Phasianidae (Galliformes). International Journal of Evolutionary Biology. 2011;2011:1–16.
